# Sampling of structure and sequence space of small protein folds

**DOI:** 10.1038/s41467-022-34937-8

**Published:** 2022-11-22

**Authors:** Thomas W. Linsky, Kyle Noble, Autumn R. Tobin, Rachel Crow, Lauren Carter, Jeffrey L. Urbauer, David Baker, Eva-Maria Strauch

**Affiliations:** 1 Department of Biochemistry, University of Washington, Seattle, WA 98195 USA; 2 Institute for Protein Design, University of Washington, Seattle, WA 98195 USA; 3 Department of Pharmaceutical and Biomedical Sciences, University of Georgia, Athens, GA 30602 USA; 4 Department of Microbiology, University of Washington, Seattle, WA 98195 USA; 5 Department of Chemistry, University of Georgia, Athens, GA 30602 USA; 6 Howard Hughes Medical Institute, University of Washington, Seattle, WA 98195 USA; 7 Institute of Bioinformatics, University of Georgia, Athens, GA 30602 USA

**Keywords:** Protein design, Molecular engineering, Molecular modelling, Solution-state NMR

## Abstract

Nature only samples a small fraction of the sequence space that can fold into stable proteins. Furthermore, small structural variations in a single fold, sometimes only a few amino acids, can define a protein’s molecular function. Hence, to design proteins with novel functionalities, such as molecular recognition, methods to control and sample shape diversity are necessary. To explore this space, we developed and experimentally validated a computational platform that can design a wide variety of small protein folds while sampling shape diversity. We designed and evaluated stability of about 30,000 de novo protein designs of eight different folds. Among these designs, about 6,200 stable proteins were identified, including some predicted to have a first-of-its-kind minimalized thioredoxin fold. Obtained data revealed protein folding rules for structural features such as helix-connecting loops. Beyond serving as a resource for protein engineering, this massive and diverse dataset also provides training data for machine learning. We developed an accurate classifier to predict the stability of our designed proteins. The methods and the wide range of protein shapes provide a basis for designing new protein functions without compromising stability.

## Abstract (web summary)

In this work the authors provide a computational workflow for the parallel, from scratch, design of proteins to rapidly explore the shape diversity of protein folds.

## Introduction

**Fig. 1:**
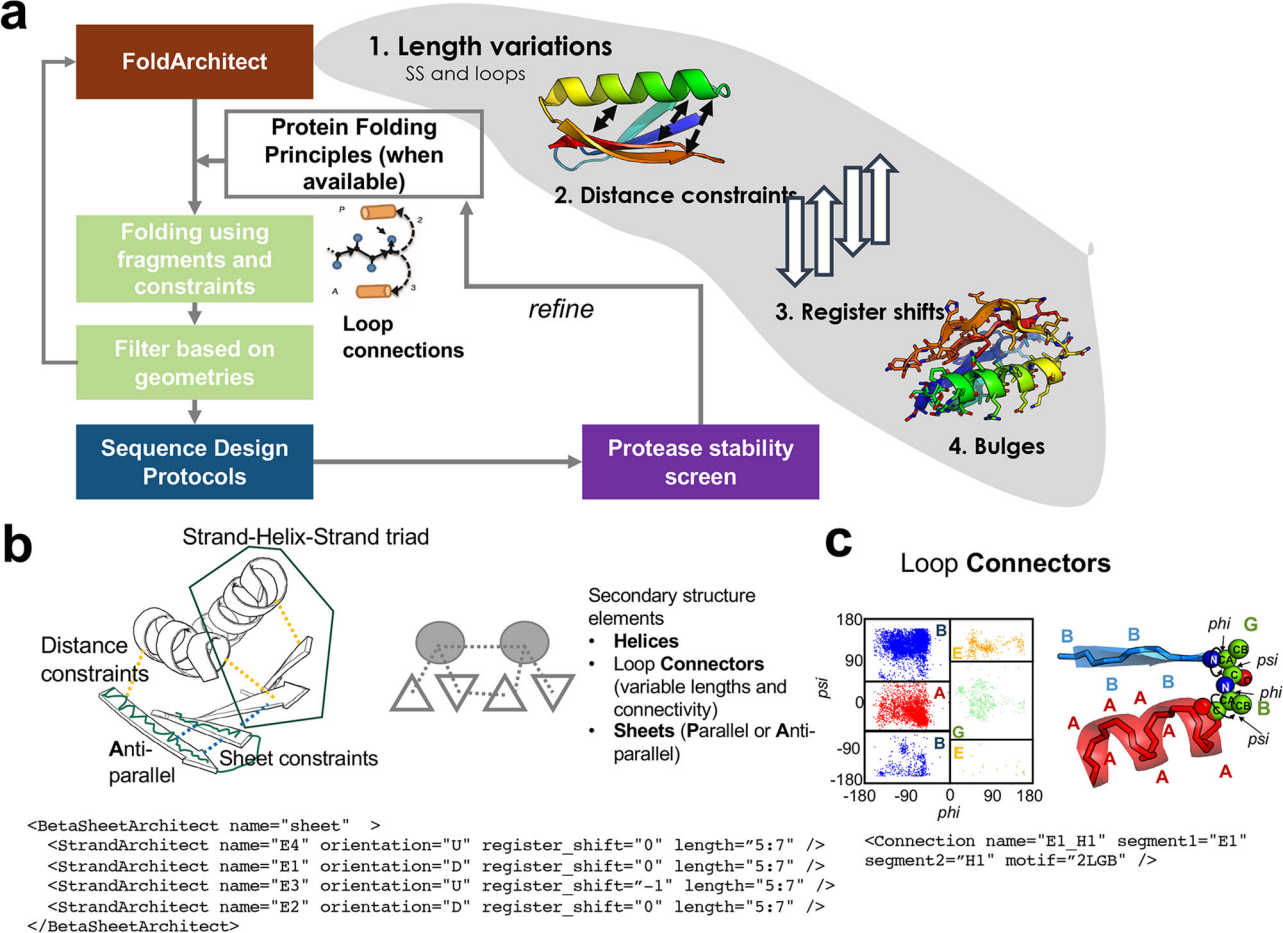
Overview of the FoldArchitect and RosettaScripts integration. **a** The FoldArchitect accepts user specification, which includes length ranges of secondary structure (SS) and loops, as well as how the distance constraints are applied (for instance, harmonic constraints). In addition, register shifts and beta bulges can be specified to introduce larger curvature into beta sheets. We then use the previously reported fragment insertion protocol^1^ and filter for geometrically realistic backbone conformations before designing the sequence of the new construct. After sequence design, the decoys are screened for their stability using varying concentrations of trypsin and chymotrypsin. The information gained from the stable designs was fed back into the FoldArchitect. **b** Various distance constraints (yellow lines) and secondary structure element pairing can be encoded in a fold-description file (following an XML format); the XML allows the user to specify next-to-length variations also, specific or ambiguous distance constraints, also pairing of helices or helix-sheet-helix, sheet orientations or bulge insertions into beta-sheets. **c** Polypeptide ABEGO regions within the Ramachandran plot can be specified for loop connections within RosettaScripts. Source data is provided as Source Data File.

Proteins are critical to most biological processes and act as catalysts, messengers, and transporters, among other tasks. Their sequences determine their structures, which define their molecular role. Yet, the natural sequence space only covers a small fraction of possible proteins^1^. The evolution of a molecular function generally occurs through the diversification of a relatively small number of known protein families. This highlights the power of shape diversity to increase functional diversity^2^. Hence, the ability to sample and control small structural variations with high accuracy represents an essential advancement in designing proteins with new functionalities. So far, a few de-novo-designed globular folds^3–5^ have been generated. Still, the structural diversity within a given fold has not been purposely sampled and experimentally verified, mainly due to the lack of a versatile computational infrastructure. Recent advances demonstrated that the exploration of a Loop-Helix-Loop motif enables geometric sampling of existing folds^6^; here, we go beyond and describe how to exhaust the plasticity of small protein folds at a large scale, sampling each secondary structure and loop connector to generate shape diversity within a given fold (Fig. 1, Supplementary Fig. 1).

Control over small geometric variations of a protein is crucial for engineering a new molecular functionality. For instance, exploring these shape variations could enable the design of ideally shaped binding proteins that fit well into a target pocket. With the advent of oligonucleotide synthesis and experimental screening technology^7^, thousands of short genes can be affordably manufactured as single oligonucleotides in large pools and screened for stability. Proteins up to 110 amino acids can also be synthesized via assembly methods^8^. Combined with yeast surface display methods, this has enabled the rapid, high-throughput experimental evaluation of the stability of thousands of proteins at a time^9^. Although machine-learning-based approaches for sampling and generating monomeric protein backbones have been recently successfully developed^10^, these methods are not designed to systematically probe the shape space of a given fold in a controlled manner. Rosetta-based computational methods for de novo design of monomeric proteins can systematically sample this space, but so far have been limited to a relatively small number of exemplar helical bundles and simple beta-strand-containing topologies. This limitation is driven by a reliance on static, predetermined secondary structure lengths and backbone torsion angles required to form the desired fold. The torsion space of canonical amino acids within a protein can be summarized into a five-letter alphabet based on the Ramachandran plot: ABEGO^11^ (Fig. 1c). This extends the secondary structure description beyond the simple alpha-helical (“A”) and beta-sheet (“B”) descriptors and provides a more detailed description of the complex loop conformational space. Design trajectories of most Rossetta de novo backbone design protocols have used predefined ABEGO sequences (as a “blueprint”) with the exception of the SEWING method, which stitches together alpha-helices^12^. In these protocols, proteins are folded in silico by inserting structured fragments curated from the PDB^3,13,14^ with the desired ABEGO sequence into an extended chain. This approach is computationally intensive and has limited scalability for larger proteins because determining the ABEGO sequence needed to fold a viable backbone requires expert intervention and multiple manually defined steps. Sampling the shape space using these methods is challenging because variations within any secondary structure lengths or loop connections require a new trajectory and blueprint file, and often require concerted and context-dependent changes to multiple secondary structures (e.g. elongation of a strand may require elongating a paired strand).

Here, we provide a computational pipeline for the massively parallel design of proteins from scratch to rapidly explore the shape diversity of protein folds and take advantage of high throughput experimental methods to evaluate it (Supplementary Fig. 1). Unlike other approaches that sample only conformation during folding trajectories, our approach also samples topology during each trajectory without prior knowledge of residue-by-residue features. It enables the design of a diverse representation of a given fold and allows sampling of (1) the lengths of each secondary structure element; (2) the distance(s) between secondary structure elements (which can also be assessed as a distribution); (3) the alignment of *α*-helices and *β*-strands and, if more than two strands are involved, their register shifts; and lastly (4) the introduction and placement of bulges to introduce curvature into *β*-strands^5^ (Fig. 1). The framework automatically applies previously discovered protein folding rules and sequence biases for loop connections^4^ and is extendable to new structural features. The RosettaScripts XML scheme allows modification of the design strategy without programming knowledge.

## Results

### Overview of the design infrastructure and folds designed

The number of amino acids encoded within a single oligonucleotide in large pools caps the number of independent secondary structure elements within each protein. For a 230 nt oligomer, the maximum length is ~64 amino acids, given that the end parts of the DNA fragments are used for homologous recombination into the yeast surface vector. This roughly allows up to about six independent secondary structure elements (meaning sheet or helix) per fold with loop connectors and their variations, which is sufficient for high throughput synthesis and screening of several diverse fold sub-families. We designed several alpha/beta (proteins are structurally composed of alternating *α*-helices and *β*-sheets in which the beta sheets are mostly parallel, Supplementary Fig. 2), alpha+beta (*α*-helices and *β*-sheets that occur separately, Supplementary Fig. 2), and several non-parametric *α*-helical folds. These included three- and four-helical bundles (3H and 4H, respectively), supercoiled 4-helical bundles (“coil”), beta-grasps, ferredoxins, thioredoxin, and two folds not seen in nature (Supplementary Fig. 3).

To create these folds, the FoldArchitect reads a fold definition and divides the fold into segments (two secondary structure elements with a loop connector) that are folded and validated incrementally. During the folding trajectory of each segment, different features, such as secondary structure length and loop types, are varied dynamically to find the best set of properties for the new segment in the context of the previously folded segments. This results in a diverse set of backbones for a given fold (Supplementary Fig. 4). For each beta-strand-containing fold, we introduced loose distance constraints (Suppl. XML files, Supplementary Fig. 5, Methods) to bias sampling toward well-formed sheets. The FoldArchitect automatically generates the beta-strand pairing between neighboring strands, and their directions (parallel *versus* antiparallel) can be specified. It uses predefined protein folding rules obtained through the design of ferredoxins^6^. As part of this work, we identified additional design rules that we have now also incorporated into the sampling of loop conformations. After in silico folding of the complete protein, we developed two different sequence design protocols. For the first protocol, we sampled rotamers of a select set of amino acids based on solvent exposure. It starts with low repulsive terms to find optimal sidechain interactions. As this scoring term increases, clashes are relieved while the strongest interactions typically remain intact. As a second protocol, we took advantage of residue “pair-motifs”. Pair-motifs are side-chain pairings observed in high-resolution crystal structures of natural proteins and have previously successfully guided the design of de novo oligomeric assemblies^15^. Before executing the same rotamer-based design approach as described for the first protocol, we first introduced paired amino acids to design the core of the proteins; this also reduced the compute time for the sequence design steps. Further, it substantially improved the rate of successful designs for 3-helical bundles (Supplementary Fig. 6). This is likely because motifs were selected heavily from larger helical protein-protein interfaces^15^. We selected designs for each topology and sequence design protocol based on a set of filter terms and their computed energies (Methods, Supplementary methods). Each design has a unique three-dimensional conformation and a unique sequence predicted to be near-optimal for that conformation (Supplementary Fig. 4).

### High throughput evaluation of designed proteins

**Fig. 2:**
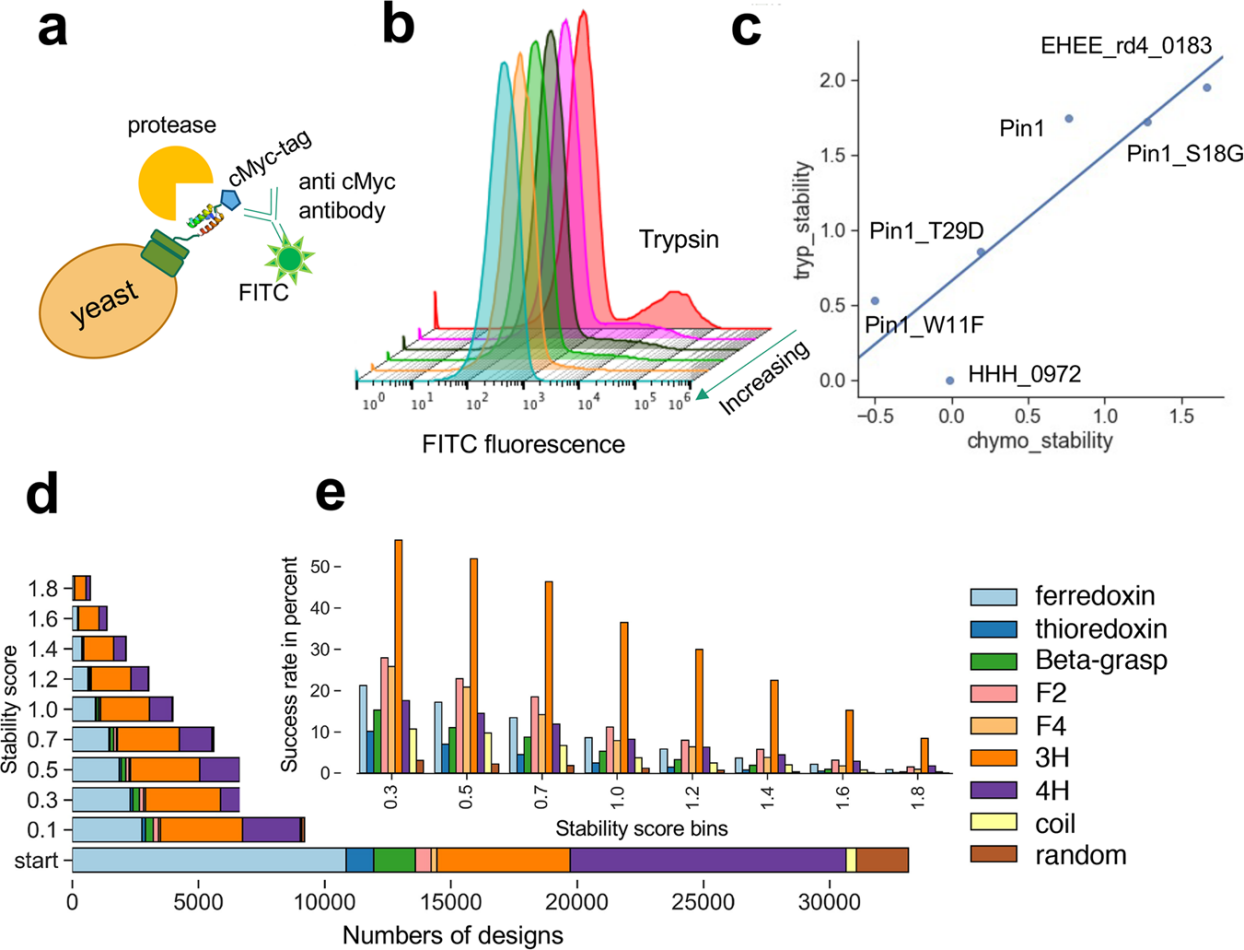
Characterization of designed small proteins using a protease-based high throughput screen on the yeast surface and biochemical analysis of individually expressed proteins. **a** Cartoon scheme of yeast surface display experiment. Unfolded proteins are cleaved and, thereby, will not be fluorescently labeled. **b** FITC fluorescence after incubation of yeast cells displaying the designed proteins as a pool with increasing concentration of trypsin; cells were labeled with a c-Myc antibody conjugated to FITC. **c** Trypsin and chymotrypsin stability of previously evaluated proteins with known stability scores were included in the pool of the query proteins as a “stability score ladder” to help adjust for protease activities. **d** The number of designs from each fold with indicated stability score or higher was used to compute **e** the “success rate” of each designed topology for a given stability score bin. Source data is provided as Source Data File.

We experimentally characterized 31,500 sequences reflecting these eight folds using yeast surface display, including about 2300 randomized sequences as negative controls. To estimate protein stability, the library of all designed proteins displayed on yeast was subjected to titrations of trypsin and chymotrypsin and uncleaved proteins were sorted into pools for each protease concentration^9^ (Supplementary Fig. 7). A direct correlation between the EC50 values of digested protein variants with their free energy was previously established^9^. Selections were performed using fluorescence-activated cell sorting (Methods); we used next-generation sequencing to count sequences from each pool. Additional selections and duplicates were also performed compared to the previously published method to improve accuracy (Methods). Using the counts obtained through sequencing, we fit EC50 values for over 31,180 sequences (including control sequences) for both proteases and calculated a stability score for each (Supplementary Fig. 8). To improve comparability between assays, we added five proteins (Fig. 2c) spanning a wide range of previously measured stability scores using the same protease-based stability evaluation procedure as a “stability score ladder” internal control. This ladder allows adjustment for the activity of each enzyme batch. Digestion of the randomized sequences enabled us to determine the stability threshold (Supplementary Fig. 9).

### Biochemical analysis of individual, soluble designs to validate high throughput screen

We randomly picked 21 designs with different topologies and stability scores for detailed characterization (Supplementary Table 1). Of these, 19 proteins expressed in *E. coli* which were then analyzed via size exclusion chromatography (SEC) to monitor the presence of aggregate, dimer, or monomeric conformations. While all-alpha-helical folds were dominantly monomeric, more dimer and aggregate formation was observed for folds containing beta-strands. All F2 and F4 designs characterized appeared to be largely in a dimeric conformation with only little of the protein found in the monomer fraction (Supplementary Fig. 10). Dimeric and monomeric fractions are not in equilibrium as they remain in the same state even after several days (Supplementary Fig. 10).

**Fig. 3:**
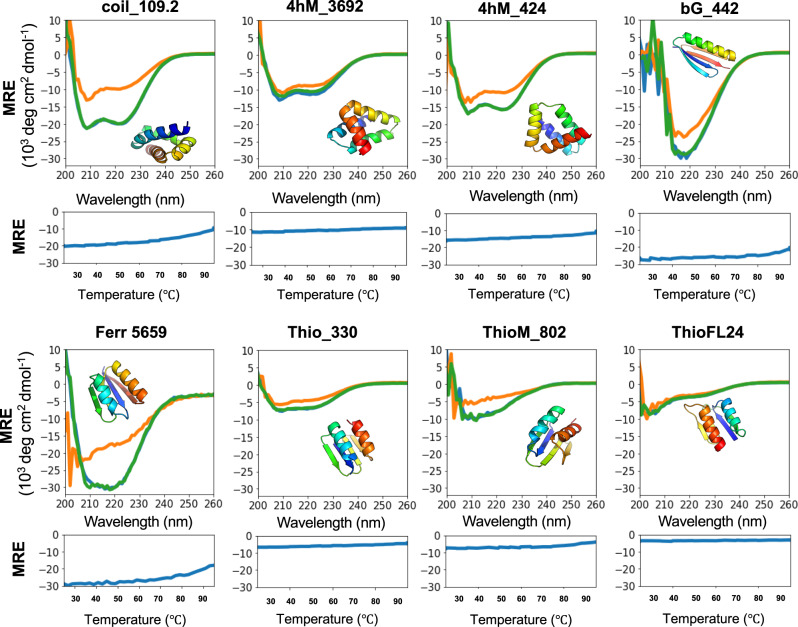
Biochemical and biophysical characterization of a subset of the designed proteins. Circular dichroism spectra were measured at 25 °C, 95 °C, and after cooling down to 25 °C (blue, orange, and green, respectively). Three measurements were averaged, and no smoothing was allowed. Melting curves show the change of molar residue ellipticity (MRE) at 220 nm at increasing temperatures. Source data is provided as Source Data File.

Using the monomeric fraction of all expressed proteins, we measured their circular dichroism (CD) spectra to assess their secondary structure formation and found all but two folded (Fig. 3, Supplementary Fig. 11, Supplementary Table 1). When comparing predicted CD spectra (using the PDBMD2CD software^16^) with experimentally determined spectra (Supplementary Fig. 11), we found that 15/19 were similar to the predictions, despite the limitations of these type of predictions. The exceptions were bGM_166, bGM_649.2, and F4M_256, which are likely misfolded, and ThioFL24, which we expected to be unfolded due to its low stability score (Supplementary Table 1). This protein was used as a control for our high throughput protease screen. It showed a high trypsin stability score, but a negative score for chymotrypsin, indicating it should be unfolded. ThioFL24 was soluble and monodisperse by SEC (Fig. 3). The CD spectrum indicated that it retained some secondary structure (Fig. 3) but was far from its prediction. This demonstrates that using two proteases is essential for this high-throughput screen. Because the monomeric version of F4M_256 was misfolded, we examined its dominant dimer fraction. We observed a secondary structure in the CD spectrum (Supplementary Fig. 11). It is possible that these folds do not have an optimized folding path and have not experienced any evolutionary selective pressure that would avoid dimerization. Further investigation would be needed to prove these assumptions. To assess whether these proteins are folded beyond their secondary structure, we measured the ^1^H NMR spectra for four designs (Supplementary Fig. 12) and confirmed they indeed formed structured conformations. We measured thermostability for eight designs (Fig. 3). We found them either to be stable at high temperatures or capable of refolding to their original folded state after being denatured (Fig. 3). We then predicted their structures using the AlphaFold2-based ColabFold^17^ interface, which allows running without the use of a multiple sequence alignment as they are de novo designed proteins (Supplementary Fig. 13). The topologies of all folds were predicted as modeled (within 0.4–3 Å), except for Thio_330 and bG-518.1. For the thioredoxin, the last beta-strand was predicted to be part of the previous helix, whereas the beta grasp fold was predicted to have a strand swap of the last two strands. AlphaFold2 currently has the highest prediction accuracy based on Critical Assessment of Structure Prediction (CASP)^17^. It is a fast and complementary structure prediction method to Rosetta-based methods and may be helpful to pre-select correctly predicted models to be evaluated experimentally. However, parameters for this should be carefully assessed in more detail and experimentally validated to identify ideal thresholds.

### Evaluation of designed fold families

The different folds varied in their success rates (fraction of designs with stability score > 0.5) using our protease digestion assay (Fig. 2). Three helical bundles generally showed the highest success rate; this is consistent with the previous report^9^. The invented folds F2 and F4 had higher success rates than the naturally occurring folds that contained alpha helices and beta-strands; thioredoxin and the super helical-coiled four helical bundles (coil) had the lowest. For the 4H and coil designs, it is possible that fewer secondary structure contacts, a smaller core, and an additional loop compared to the 3H designs may decrease the margin for error.

To explore small alpha+beta proteins, we sampled variations of the beta-grasp (Supplementary Fig. 14) and variations of the ferredoxin (Supplementary Fig. 15) fold. Previous attempts to design very small versions of the ferredoxin fold have failed, resulting in unfolded proteins^4^. We extensively sampled secondary structure lengths and registers for the ferredoxin fold and found that there are indeed geometric limitations (Supplementary Fig. 15), likely due to the inherent requirement of right-handed strand-helix-strand motifs. We were able to design small and stable variations of this fold as small as 55 amino acids in length (Supplementary Fig. 15E).

Parametrically de novo designed helical bundles^18^ or repeat proteins^19^ are highly stable. However, the non-parametric small helical bundle fold space can provide more diverse shapes which have not been extensively sampled, particularly the 4H bundles. To address this, we designed a variation of 4H bundles, “coil” designs, that alternate between shorter and longer helices, using specific distance constraints to guide supercoiling. Furthermore, protein design rules for the loop connections of two alpha helices and angle variations were missing up to this point; having revealed these with our 3H and 4H bundles, we implemented these connector rules to our design platform (Supplementary Figs. 16–18). To further test our design algorithm, we explored alpha+beta folds beyond those that are naturally occurring. We derived *αββββα* and *βααβββ* folds, named F2 and F4 (Supplementary Fig. 3). F2/4 designs expressed well and showed distinct peaks using SEC. Although there were aggregates or higher order oligomers present, the designs appeared to be mostly dimeric after expression in *E. coli* and Ni-NTA purification (Supplementary Fig. 9). This suggests a mechanism at work that is not captured by our design method; it is possible that the lack of interconnectedness in these folds enables the fold to become a swapped dimer. The thioredoxin fold, for example, contains a *β*2 - *β*3 strand swap that interweaves the beta strands of the thioredoxin fold and may prevent the formation of domain swapped dimers. Folds that have a direct linear connectivity of their secondary structure elements, such as F2 and F4, may require further optimization through disulfide connections or, perhaps, negative design that discriminates against a swapped dimeric conformation. Although the oligomeric state was not as designed, our pipeline was readily able to produce models for these folds that are stable against proteolysis and are folded.

To evaluate shape diversity, we compared both protease-stable and unstable proteins for each fold. We compared distances between secondary structure elements, register shifts, dihedral of the outer beta-strands to describe the curvature of the sheets, dihedrals between adjacent secondary structure elements, interhelix distance, and helix angles given specific *phi* and *psi* angles in their loops, demonstrating that each fold has local plasticity, validating our basic rules of de novo protein design and suggesting an additional set of rules (Fig. 2, Supplementary Figs. 14–18).

### De novo design of a minimized thioredoxin fold

**Fig. 4:**
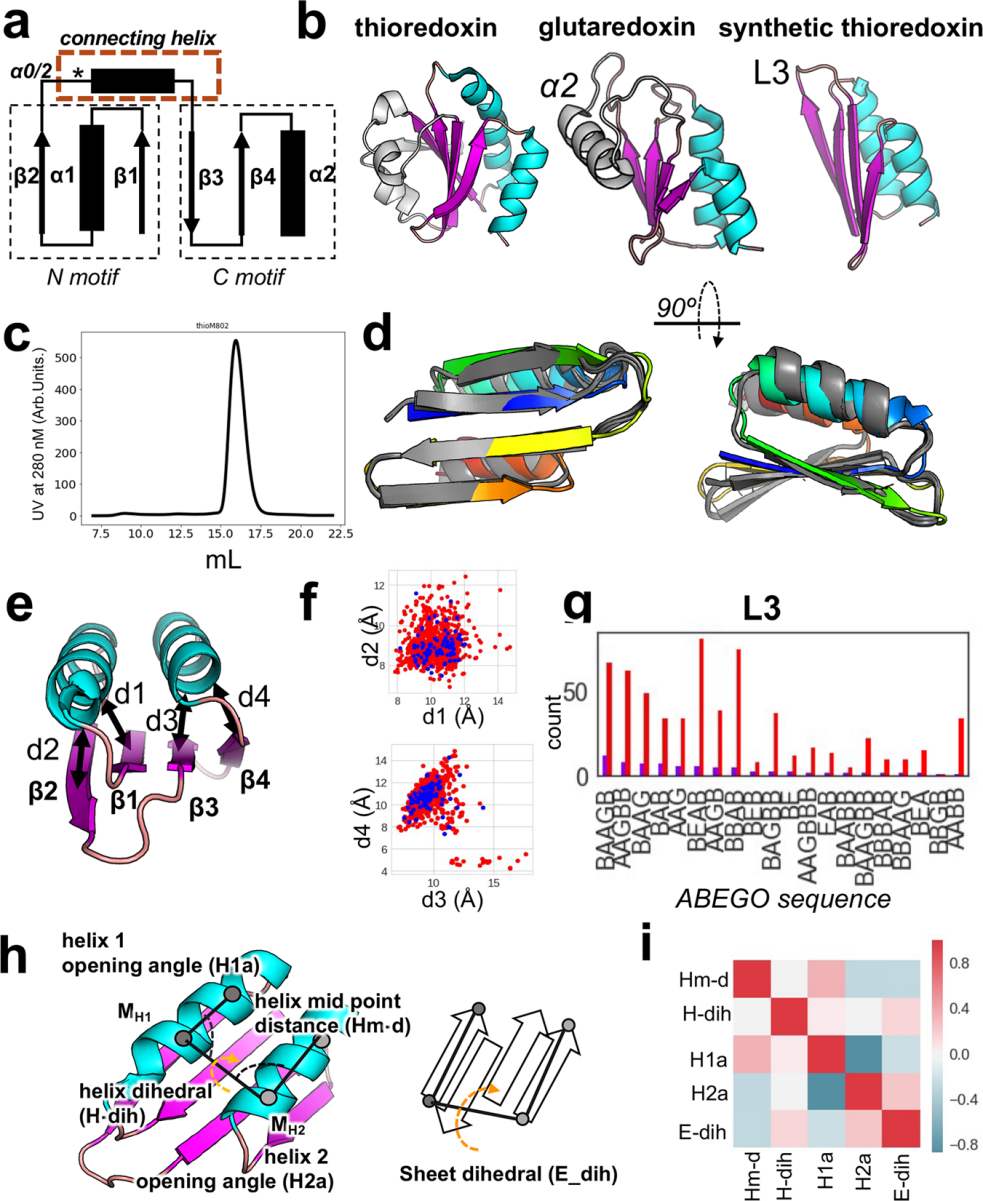
Synthetic thioredoxin fold. **a** Connectivity of the basic thioredoxin fold showing N and C motifs; the N motif (*βαβ* element) represents the structural feature that allows domain extensions. The commonly found *α*0, also called *α*2, was replaced with the L3 loop for a minimized thioredoxin fold. **b** Thioredoxin folds found in nature (left and middle) and the designed synthetic thioredoxin (right). **c** SEC of ThioM_802 shows a single defined peak using a Superdex S75 column. **d** NMR structure (grey) ensemble (PDB 7LDF) compared to the model (rainbow coloring from N-terminus (blue) to C-terminus (red) of Thio_802. The His-tag was omitted. **e** Distances in Å, measured between secondary structure elements. **f** Correlation of distances in stable (blue) and unstable (red) designs. **g** Several occurrences of L3 loop variations (represented by their ABEGO sequences) in unstable (red) and stable (blue) designs. L3 replaces the *α*2 helix of natural thioredoxins; several conformations allow a stable protein. **h** Definition of geometric descriptions measured to evaluate shape diversity, including how the sheet dihedrals were calculated. **i** Interdependence of structural factors, such as the helix midpoint distances (Hm-d), helix dihedrals (H-dih), helix 1 and 2 opening angles (H1a and H2a), reveal several correlations within a fold. Red indicates correlated, whereas blue shades indicate a negative correlation. Source data is provided as Source Data File.

So far, de novo design of a thioredoxin fold had not been reported. Here, we tackled the even more challenging problem of minimizing the overall fold. Naturally occurring thioredoxin has a three-layer *β/α* sandwich with the central sheet formed by five strands flanked by two *α*-helices on each side (Fig. 4). Many thioredoxin-like proteins have variations in their *α*-helices or the fifth *β-*strand (Fig. 4a, b). The conserved core elements of the thioredoxin fold can be subdivided into an N-terminal *βαβ* motif and a C-terminal *ββα* motif (Fig. 4a), which is commonly connected by a small helix (*α0* or *α2*, Fig. 4a, b). The *βαβ* element (characteristic for the alpha/beta family) is found in many larger proteins as it is the connecting motif that enables the expansion of the protein domain space^2^ and distinguishes members of this superfamily from the alpha+beta family which does not have the repetition of this motif. Its incorporation will generally allow for extensions of a fold and thereby provides means to build larger proteins. Hence, the ability to design this element with high shape diversity provides a tool for building larger and more shape-diverse folds and domains. We designed a minimal version of the thioredoxin fold, containing only the core four sheets and two parallel or anti-parallel helices, replacing the common *α2* helix with an extended loop (Fig. 4g). We solved the structure of one of our designs using nuclear magnetic resonance (NMR, Fig. 4d, Supplementary Fig. 19, Supplementary Table 2). The NMR ensemble agrees with the designed model with the top structure having an RMSD of 1.9 Å (based on C-alpha atoms) compared to the model; deviations are mostly from the last helix. All *β-*strands were close to the design model (1.1 Å C-alpha RMSD) making this the first accurately de novo designed thioredoxin fold.

### A single machine learning model to identify stable designs

**Fig. 5:**
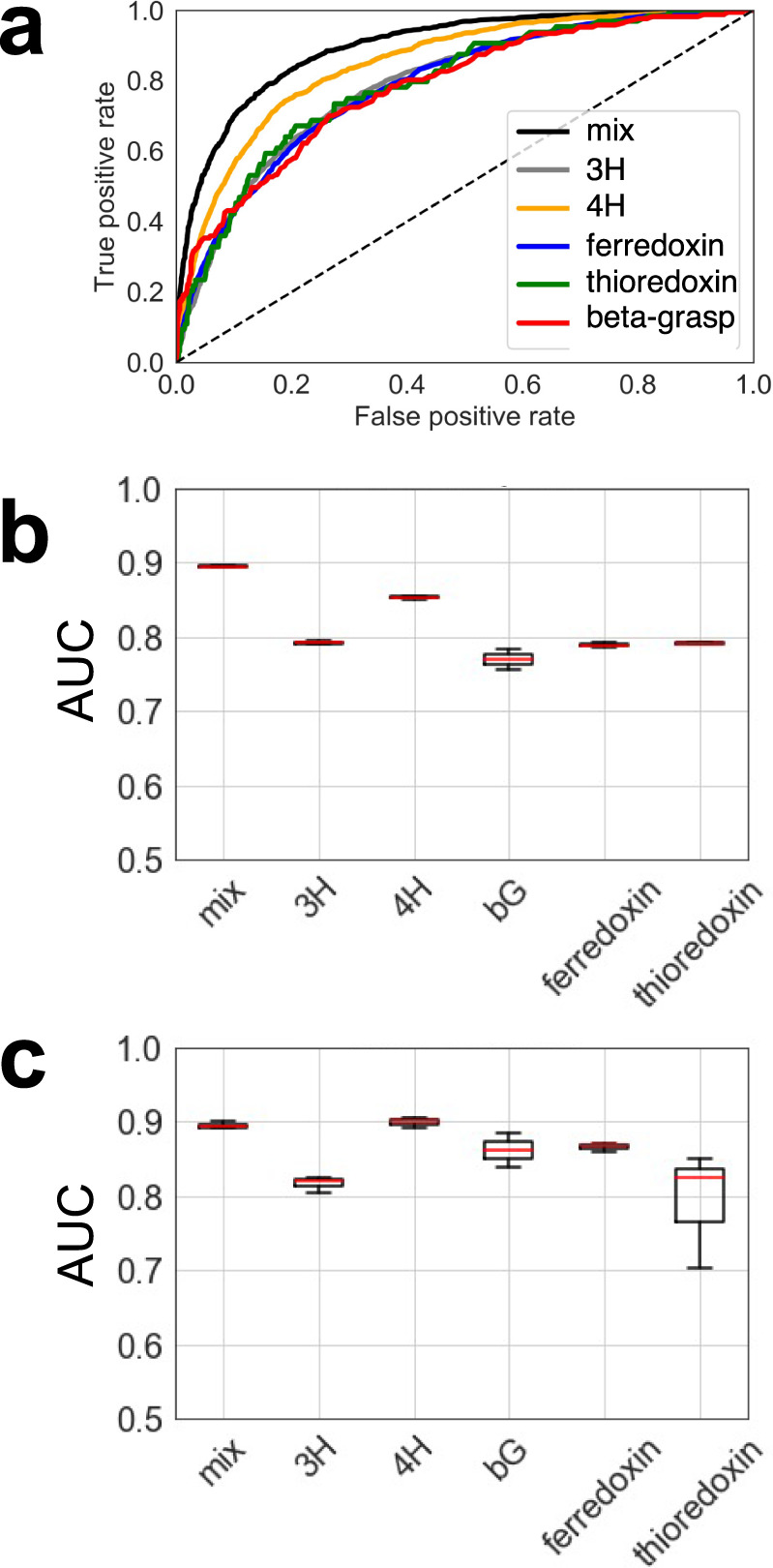
Performance of the stability prediction classifier. **a** The receiver operator curve (ROC) illustrates the predictive power of our Random Forest classifier. To avoid potential bias, we compared the ROC of predicting stabilities for any protein within the whole set vs. a ROC of the different folds or all combined (mix) after training on the other folds within the set. For this prediction, one fold at a time was omitted (“dropout”), and the classifier was trained on all other folds to predict the stability of the unseen fold. **b** AUCs of three iterations of the predictions described under (A); This graph represents the minimum, maximum, and median in the data set. **c** Summary of AUCs of predictions for individual folds. Boxplots describe the spread of data after three independent training and predictions, showing minimum, maximum, and median. Source data is provided as Source Data File.

Having a large and diverse set of stable and unstable proteins of different folds available with varying shapes and physical properties, we were able to re-evaluate stability-defining features and develop a classifier based on a Random Forest model^20^ that determined whether a given small protein is stable with high accuracy. Compared to previously described physical and statistical features for stability, we evaluated additional features describing residue interaction networks and energetic contributions of individual amino acids within tightly connected hubs of residues, resulting in a total of 110 sequence- and structure-based features. The most predictive of these additional features was the overall energy contributions of the most connected residue: residues that contact many other residues are interaction hubs. They tend to be generally highly buried and provide the “glue” of the hydrophobic core of proteins. Hence, favorable energetics of these “hub” residues is essential for the protein core formation while potential clashes could result in instability. As previously seen^9^, correct local geometry as measured through alignment with short structural fragments (Supplementary Figs. 20–23) is the most predictive feature for the folding of a de novo designed protein, followed by the number of hydrophobic residues within the protein core. Unlike previous studies, by using our larger and more diverse fold data set and descriptive features, we were able to train a model on multiple folds, instead of one fold at a time^9^ (Supplementary Fig. 20) and could predict stability even for unseen folds (Fig. 5c, Supplementary Figs. 21–23). We believe that this diverse scaffold set is enabling us to learn more general descriptions of these folds and increases the predictive power of the model. To confirm this observation, we predicted the stability of the previously published data set with several different small folds by Rocklin et al.^9^ and observed a predictive power of an AUC of around 0.83 using our single classifier (Supplementary Fig. 23).

## Discussion

While previous work simulated the space of a handful of folds^3–5^ using the elaborate blueprint-based protocols combined with manually defined multi-step for the assembly of larger folds, only a few designs were experimentally verified in solution, with the exception of mini-proteins that were examined in the context of the development of the protease-based high throughput screen. We built upon previously discovered protein design rules^4^, including connection rules for strands and helices, and provide a versatile fold assembly and design pipeline that allows dynamic sampling of a given fold during the in silico folding trajectory. Our extensive sampling and high throughput evaluation allowed us to examine thousands of designs at once, revealing geometric diversity of different folds. We also extracted additional rules for protein design, such as loop connectors for helical elements, which we in turn incorporated into the design algorithm and can be readily accessed in any Rosetta design step. Lastly, our extensive study allowed us to develop a simple prediction model to help future design approaches identify stable proteins. The algorithm is implemented into the RosettaScripts^21^ framework, which enables all design features and protocols to be accessed and executed in the form of XML files without prior programming knowledge. This work also provides an extensive scaffold database as a general resource for alternative scaffold engineering and protein design projects, which recently resulted in the development of picomolar COVID-19 inhibitors^22^ and also served as starting scaffolds for a variety of newly designed binding proteins targeting FGFR2, TrkA, IL7Ra, and VirB8 for which co-crystal structures illustrate atomic accuracy of the overall folds^23^.

## Methods

### Computational protein design

#### Overview

Proteins were designed using three steps. First, the backbones were constructed, which outlay the three-dimensional structure of the final fold. This step made extensive use of the here developed pipeline and differs from previous approaches that have utilized the blueprint builder. The second step involves sequence design for which we utilized two different protocols. The third step is the selection of designs to test. All steps can be done in the RosettaScripts^21^ XML format and our protocol has been deposited on github [https://github.com/strauchlab/scaffold_design].

#### Backbone design

The underlying algorithm of the Fold Architect (FoldArchitectMover) has several modular components that work together to design a de novo peptide backbone for a fold. Together, these components provide the framework to take a fold-level description (e.g., 3 helix bundle, each helix of length 10–15 a.a. connected by loops of length 3–4 a.a.) and produce protein backbones with the desired secondary structure, realistic geometries, and helix-pairing interactions. These modules are part of different “sub-architects,” which uses a user-provided (via XML) description of a fold or subset of residues within a fold to create a set of per-residue instructions; a “pose folder” that applies an in silico folding algorithm, which uses the instructions provided by the architect to perform the folding process; a set of “filters”, which can be any Filter recognized by Rosetta, which evaluate the backbone generated by the pose folder to ensure that it is correctly folded; and a “perturber”, which instructs an architect to generate a set of instructions in the event that the filters did not accept the backbone. Each of these components can be arbitrarily extended to support new algorithms by creating subclasses of the Architect, PoseFolder, Filter, and Perturber classes.

One complication with the de novo extended chain folding of backbones is that it scales poorly with length; a single missing hydrogen bond in a backbone can lead to incorrect secondary structure and failure of filters. While a helix of length 15 might be correctly formed after most fragment insertion as part of Monte Carlo trajectories, a complete 40–50 amino acid long miniprotein fold might require thousands of trajectories from an extended chain to find one that is correctly folded. This has been addressed in previous work with single folds through extensive intervention by an expert. To address this problem for arbitrary folds, we developed an algorithm (DivideAndConqueror) that identifies subsegments of a full-length backbone that can be folded individually and generates a strategy to build the backbone incrementally, piece-by-piece. The DivideAndConqueror algorithm uses the architect(s) to split the work for a full backbone into subsegments as small as possible that contain a complete pairing; all possible divisions are considered. For example, for a simple fold with topology ββαβ (three antiparallel strands + one helix that is paired to the strands), the algorithm might divide the work by first folding ββ (contains a pairing between the two strands), then adding α (contains a pairing between the helix and already-folded strands), and finally β (contains pairing to an already-folded strand). Each subsegment is folded with the modules described above, and once built, is evaluated using the filters. If the model passes the filters, the algorithm then preserves the backbone and folds the next structural element; if the model fails the filters, the Perturber instructs the architects to permute the parameters of the subsegment (e.g. secondary structure lengths, register shift, *phi* and *psi* angles, loop connectivity) and another folding attempt is made. In this way, the FoldArchitect gradually adds and folds a backbone while sampling different lengths, ABEGO combinations and other parameters that fit within a combination of user-defined restraints, as well as a series of previously discovered protein design principles to find parameters required for correct properties of the fold.

As there are five distinct structure-related areas of *phi*-*psi* angle distributions, proteins can be described with a five letter alphabet^6^: ABEGO (Fig. 1c). This extends the secondary structure description beyond the simple alpha-helical, beta-sheet and loop region, yet narrows the description of the loop conformational space which is necessary to guide the in silico folding process. We eliminate reliance on a single, immutable linear AGEGO sequence in form of a “blueprint” file or possibly a handful of blueprints in favor of the abstract description of a fold described here.

Folds were in silico folded by inserting structured fragments curated from the PDB^6,7^ (Supplementary Fig. 1) specified by the architect-determined ABEGO sequence into an extended chain of poly-valine residues.

#### Loop connection sampling

Instead of sampling all possible loop conformations for a given loop length, only loop conformations commonly found between αβ, βα and ββ connections as previously discovered were sampled^4^. In addition, our study identified rules for the connection of helical elements, which we also incorporated. As more principles are identified, they can be readily added. Furthermore, we enabled the possibilities to provide distance constraints between adjacent elements and incorporate “bulges” to introduce curvature into a strand^5^, which we took advantage of to build beta-grasps. Previously discovered protein design principles for loop connections are respected and the protein is dynamically assembled adding one segment at a time.

#### Secondary structure element pairings

Secondary structure elements that interact with one another in the desired fold are identified by “Pairings.” Movers and filters can then also use this information to obtain information about the desired fold. The different pairing types are “HelixPairing”, which describes a pairing between two helices (e.g., parallel/antiparallel); and “StrandPairing”, which describes a strand-strand pairing (e.g., parallel/anti-parallel, register shift); and “HelixSheetPairing”, which describes an interaction between a helix and a beta-sheet.

#### BetaSheetArchitect

The BetaSheetArchitect is used to define de novo beta-sheets by combining information from StrandArchitects. Sheets are defined spatially by looking at the face of the sheet (this does NOT use N– > C ordering). Strands are assumed to be paired to the strands that are defined above/below.

The architect automatically adds the appropriate strand pairings. The architect will only attempt to build valid sheets, those where the fully built sheet has no unpaired residues.

#### Distance constraints

Distance constraints of any kind can be applied ambiguously without knowledge of the final length used in the dynamic algorithm. We developed a mover that allows the application of distance constraints between residue selectors (DistanceConstraintGenerator). Because folds are built dynamically, key features are not known prior to the assembly process, such as the size and the N or C-terminal residues for each secondary structure element. To address this, we developed a NamedSegment residue selector that keeps track of each element and tracks residue numbering as needed. This allows the assignment of distance constraints even without knowing precisely the final structural composition of the fold. Distance constraints were coupled to a simple harmonic or a bounded harmonic function with a loose standard deviation of 2 Å. After measuring distances between helices and sheets of a few example beta-grasps and ferredoxin folds, we chose 8 Å as the default starting value for sheet and helix distances. Adjustments were made depending on the output for a given fold and distances were increased or decreased. For example, in the beta-grasp fold, the distance between the C-terminal part the helix and the N-terminal part of the second sheet was adjusted to 9 Å. Details are within each XML file for the computed folds.

#### Helix “kink”

This filter monitors the curvature of helices and allows to restrict them. We generally only allowed bending of less than 15°.

#### Backbone design

To build the tertiary structure of a protein, short, structured fragments with the desired ABEGO sequence are used for its assembly. However, recording a tertiary structure as a single ABEGO sequence in an individual file does not allow for variation in lengths of any secondary structure element, register shifts, bulges, as well as distant constraints during the trajectory. The residues involved in the latter would also need to be precomputed based on each blueprint. Further, structures that are longer than 40 amino acids would require two independent folding steps to avoid large amounts of time spent sampling the possible backbone conformations within a given ABEGO space. Thereby the infrastructure was limiting to large-scale design of highly diverse scaffolds of a desired fold.

We addressed these limitations by creating a definition for a fold with parameters that can be altered during a trajectory. For the design of a single protein of a given fold, a fold description is read and subsegments are incrementally varied, built and computationally validated. Folding of the backbone was followed as previously reported through fragment insertion, with the difference that a subsegment was folded at a time enabling efficient sampling within the pre-defined parameter space. The Perturber adjusts dynamically the folding process (for instance by varying lengths or type of loop insertion, register shift) to satisfy all specified parameters of the folded backbone.

#### Sequence design

Backbone constructs representing the complete fold were stringently filtered for the *omega* and *rama* angles before designing their sequence. Two different design protocols were utilized, one utilizing the previously described pair-motifs^1^ to design the core of the proteins. The pair motif database contains two directly interacting side chains of two amino acids extracted from crystal structures, thus describing a “pair”. We observed that using this protocol, efficient sequence design was observed that passed all filters, such as local structural geometry, the average degree of connectivity within a certain radius to ensure good packing, Rosetta scores, and several others (See SI and code availability).

#### Scoring matrix and design selection

All score terms for filtering and evaluating designs are summarized in the SI. Their implementation can be found in the design protocols sequencedesign.xml and sequencedesign_w_motifs.xml, rescore15.xml and rescore16.xml followed by adding terms previously reported as “enhanced_score.sc”^9^. For each fold, 2000–12,000 finished design models were generated and scored.

### Random forest prediction model

Models were fit using the scikit.learn package^20^ using the Anaconda2 package, Python 2.7.16 (default, Sep 24 2019). A random forest classifier model was generated using listed 110 features using fivefold cross-validation to evaluate its accuracy. We used 500 as numbers defining the decision trees for the estimator and the default “gini” as criterion over entropy. The Gini index prefers the features possessing the least value of the Gini Index while building the trees. The Gini Index is determined by deducting the sum of squared of probabilities of each class from one (1):

1 $${{{{{\rm{Gini}}}}}}\; {{{{{\rm{Index}}}}}}=1-\mathop{\sum }\nolimits_{i=1}^{n}{({P}_{i})}^{2}$$

We allowed bootstrapping and used the “out-of-bag” samples to estimate the generalization accuracy. For each tree, the whole dataset is used.

### AlphaFold2 predictions

Structure prediction of analyzed scaffolds was performed using the ColabFold batch mode. The single sequence option was utilized as multiple sequence alignments for de novo scaffolds are not applicable. The unrelaxed prediction output structures were then repacked and minimized in Rosetta. Ca Rmsd of original designs with the AlphaFold2 predictions were computed based on the relaxed structure.

### Library generation

Amino acid sequences of designed proteins were encoded into DNA using DNAworks2.0 and “ecoli2” codons^24^. Oligo libraries encoding designs and control sequences were purchased from Agilent Technologies as part of a 27,000-oligonucleotide pool. For genes shorter than 230 base pairs, additional amplification sequences were added as previously reported^2^ to amplify sequences equally. Amplification was performed using a qPCR (BioRad) to avoid overamplification. The number of cycles was chosen based on a test qPCR run to terminate the reaction at 50% maximum yield. Second, this reaction product was gel extracted to isolate the expected length product and re-amplified by qPCR to obtain larger amounts. The amplified PCR product was gel extracted and concentrated for the transformation of EBY100 yeast^25^ (1–2 µg of insert and 1 µg of a linearized vector). Yeast display employed the pETCON3^26^, which was linearized by digesting its DNA with *NdeI* and *XhoI*. The amplified libraries included 40 bp segments on either end to enable homologous recombination with the pETCON vector. Gel extraction and PCR purification were performed using QIAquick kits (Qiagen Inc).

### Yeast display proteolysis

Protease reagents Trypsin-EDTA (0.25%) solution was purchased from Life Technologies and stored at stock concentration at −20 °C. α-Chymotrypsin from bovine pancreas was purchased from Sigma-Aldrich as lyophilized powder and stored at 40 µM concentration in TBS (20 mM Tris 100 mM NaCl pH 8.0) + 100 mM CaCl_2_ at −20 °C. Each reaction used a freshly thawed aliquot of protease.

EBY100 yeast cell cultures were induced for 16–18 h at 30 °C in SGCAA^27^. Induced cells were digested with increasing concentrations of chymotrypsin and trypsin in separate tubes. Cells were normalized to 1 mL at O.D. 1 (12–15 Mio. cells), washed, and resuspended in 250 μL PBS (20 mM NaPi 150 mM NaCl pH 7.4) for trypsin reactions, or TBS for chymotrypsin reactions). Proteolysis was initiated by adding 250 μL of room temperature protease in buffer (PBS or TBS), followed by vortexing and incubating the reaction at room temperature (proteolysis reactions took place at cell O.D. 2).

The library was assayed at five protease concentrations over sequential selection rounds, as summarized in the experiments.csv file. For trypsin digestions we used 0.07 μM, 0.21 μM, 0.64 μM, 1.93 μM, and 5.78 μM protease; chymotrypsin assays used 0.08 μM, 0.25 μM, 0.74 μM, 2.22 μM, and 6.67 μM protease. Selections at lower concentrations (selection strength 1−3) of each protease were performed starting from the freshly transformed and induced yeast library. Following these selections, higher concentration conditions were performed as indicated in the *experiment.csv* file. This file contains the precise order of selections, including cells sorted and selected. Selection strengths (as indicated under “parent”) reflects above listed concentrations respectively; parent 0 represents the starting library pool, whereas 1 reflects the lowest concentration as listed above (0.07 μM for trypsin and 0.08 μM for chymotrypsin. The highest protease concentration was parent 5.78 μM for trypsin indicated as selection strength 5. These data are included in the file *experiments.csv* and were used in the EC_50_ fitting procedure.

After 5 minutes, the reaction was quenched by adding 1 mL of chilled buffer containing 1% BSA (referred to as PBSF or TBSF), and cells were immediately washed 4x in chilled PBSF or TBSF. Cells were labeled with anti-c-Myc-FITC for 10 minutes (5 µL in 100 µL PBSF), washed twice with chilled PBSF.

### Fluorescence activated cell sorting

Labeled and washed cells were sorted using a Sony SH800 (software version 2017) flow cytometer using the “Ultra Purity” settings. Events were initially gated by forward scattering and backward scattering area to collect the main yeast population and then by forward scattering width and forward scattering height to separate individual and dividing cells (which were used for analysis) from aggregating cells (Supplementary Fig. 7). Following these gates, cells were gated by fluorescence intensity in one dimension (Fig. 1B). Small adjustments were made to this gate based on daily conditions to maximize the separation between the major displaying and non-displaying populations. Post-sorting analysis was done in FloJo (v. 8 and 10.8). For each sort, we recorded the fraction of cells passing the fluorescence threshold before proteolysis (using cells from the same starting yeast population but untreated with protease) and after proteolysis and recorded the total number of cells collected for each condition. Generally, about 10 million cells were sorted for each protease concentration including the control.

### Next-generation sequencing and processing of raw deep sequencing data

Plasmids of sorted and unsorted populations were extracted using the Zymo prep kits (Zymo, version 2) of yeast cultures containing the pETCON3^26^ with some modifications. After DNA extraction, the prep was digested with Exo1 and Lambda exonuclease (NEB). Cells were frozen at −80 °C before and after the zymolase digestion step to promote efficient lysis. One-half of the plasmid yield from the Zymoprep was used as the template for the first PCR amplification. Illumina adapters and 6-bp pool-specific barcodes were added in the second qPCR step^26^. Unlike libraries prepared for transformation, DNA prepared for deep sequencing was gel extracted following the second amplification step. The DNA was pooled and sequenced using a mid-size kit on a NextSeq (Illumina) sequencer. Each library in a sequencing run was identified via a unique six bp barcode. Following sequencing, reads were paired using the PEAR program^28^. Reads were considered counts for a particular ordered sequence if the read (1) contained the complete *NdeI* cut site sequence immediately upstream from the ordered sequence, (2) contained the complete *XhoI* cut site sequence immediately downstream from the ordered sequence, and (3) matched the ordered sequence at the amino acid level.

### EC_50_ estimation from sequencing counts

To determine protease resistance from our raw sequencing data, we used the previously reported probabilistic model to calculate maximum a *posteriori* estimates of the protease EC_50_ of each member of the pool. It assumes that proteolysis (i.e., any cleavage that results in detachment of the epitope tag) follows pseudo-first-order kinetics, with a rate constant specific to each sequence. Scripts were used precisely as previously reported without modification^9^ and directly taken from the reported repository https://github.com/asford/protease_experimental_analysis.

### Expression of individual proteins, purification, and characterization

Genes for selected design for detailed biochemical evaluations were cloned into pET29b+ and expressed in Lemo21 cells (DE3) (NEB) supplemented with 50 ug/mL kanamycin either using Studier autoinduction media^29^ or at 18 °C in Terrific Broth (TB) media using 1 mM IPTG for a 3–4 h induction at an O.D. of 0.7. Briefly, starter cultures were grown overnight at 37 °C TB medium with added antibiotic and used to start a 500 mL culture at a 1/50 dilution. His-tagged proteins were purified using a nickel column purification step (QIAGEN). Following IMAC, designs (labeled and unlabeled) were further purified by size-exclusion chromatography on ÄKTA pure (GE Healthcare) using a Superdex 75 10/300 GL column (GE Healthcare) in PBS. The monomeric fraction of each run (typically eluting at the 15 mL mark) was collected and immediately analyzed by circular dichroism far-ultraviolet (CD) measurements were carried out with the Olis DSM 1000 CD Spectrometer except for the ferredoxin folds reported here, which were measured with an AVIV spectrometer (model 420) CD Spectrometer. For Olis data, a custom python script was used to decode the binary files and plot the data. Wavelength scans were measured from 195 to 260 nm at 25 and 95 °C. Data below 200 nm showed increased noise levels and was therefore not plotted. Temperature melts monitored dichroism signal at 220 nm in steps of 2 °C/minute with 30 s equilibration time. Wavelength scans and temperature melts were performed using 0.35 mg/ml protein in PBS buffer with a 1 mm path-length cuvette, protein concentrations were adjusted accordingly.

One-dimensional 2H NMR spectra were acquired for samples of four of the proteins ranging in concentration from ~0.12 to 1.0 mM at 25 °C using the instrumentation described below.

Protein concentrations were determined by absorbance at 280 nm measured using a NanoDrop spectrophotometer (Thermo Scientific) using predicted extinction coefficients. Protein concentrations for designs lacking aromatic amino acids were measured by Qubit protein assay (ThermoFisher Scientific).

### Isotope labeling for NMR

The expression of uniformly ^13^C, ^15^N-labeled ThioM_802 protein for NMR analysis utilized M9 media with ^13^C glucose and ^15^N ammonium salts (Sigma) as the sole carbon and nitrogen sources, respectively. A 30 mL overnight culture was grown and used to induce a 500 mL M9 culture. At an O.D. of 0.5, cells were induced with 1 mM IPTG and grown overnight at 18 °C. Purification was performed as described above.

### NMR spectroscopy and solution structure determination

All NMR spectra were acquired using a Varian INOVA instrument operating at 600 MHz (^1^H). After Ni-NTA purification, proteins were run over SEC using the same column as above but using a lower salt buffer (10 mM Phosphate buffer, 50 mM NaCl, pH 6.85). Samples were concentrated to about 607 µM using a Amicon Ultra-4 (Millipore), which may introduce trace elements of glycerol as indicated in its manual. A total of 5% D_2_O was added. The temperature of the sample was maintained at 25 °C. For structure determination, a single sample of uniformly ^13^C, ^15^N-labeled ThioM_802 protein was used for all experiments. The sample volume was approximately 300 uL in a susceptibility-matched NMR tube (Shigemi Inc.). Chemical shifts were referenced in the recommended manner using an external, standard sample of Na^+^DSS^-^ in D_2_O^30^. Data were processed and analyzed using Felix NMR.

The main chain, and some side chain, chemical shifts of ThioM_802 were assigned using established triple resonance approaches employing a standard suite of experiments ((^1^H, ^15^N)-HSQC, (^1^H, ^13^C)-HSQC, HNCA, HN(CO)CA, HNCACB, CBCA(CO)NH, HNCO, HN(CA)CO, HBHA(CBCACO)NH). The remaining side chain resonances were assigned using TOCSY, NOE, and aromatic-specific experiments (HCCH-TOCSY, HCCH-COSY, H(CCO)NH-TOCSY, C(CO)NH-TOCSY, (HB)CB(CGCD)HD, ^1^H-TOCSY relayed constant-time (^1^H, ^13^C) HMQC (aromatics), NOESY-(^1^H, ^15^N)-HSQC, NOESY-(^1^H, ^13^C)-HSQC, NOESY (^1^H, ^13^C)-HSQC (aromatics)). The NOESY experiments were acquired with 100 ms mixing times.

Manually identified peaks/signals in the 3D NOESY spectra were assigned and calibrated, distance restraints defined, and initial structures determined, in an iterative manner, using CYANA 2.1^31^. Using TALOS-N^32^ main chain dihedral angle restraints, secondary structure predictions were derived based on assigned chemical shifts. These structural restraints were used to calculate a set of 100 initial structures starting from an extended structure using simulated annealing protocols in CNS 1.3^33^ software suite for macromolecular structure determination^33,34^. These were then refined, and the 20 structures with the lowest energies were selected as the final structural ensemble. CNS, MOLMOL^35^, PyMOL, PROCHECK_NMR^36^, and PROMOTIF^37^ were used to analyze the ensemble and generate molecular models. The Protein Structure Validation Software Suite (PSVS, https://montelionelab.chem.rpi.edu/PSVS) and the MolProbity server^38^ were also used to analyze the structures. Chemical shifts, structural restraints, and atomic coordinates have been deposited in the BMRB (entry 30844) and the PDB (PDB ID 7LDF).

### Reporting summary

Further information on research design is available in the Nature Portfolio Reporting Summary linked to this article.

## Supplementary information

Supplementary Information

Reporting Summary

## Data Availability

The NMR structure, experimental restraints, chemical shifts, and related information have been deposited in the Protein Data Bank (PDB) under accession code 7LDF and in the Biological Magnetic Resonance Bank (BMRB) as entry 30844. Sequencing data summary, stability and score files are available from github [https://github.com/strauchlab/scaffold_design]. Scaffolds and next-generation sequencing raw data can be sent upon request. Source data is provided with this paper. Source data are provided with this paper.

## Source data

Source Data

## Code availability

The Rosetta license can be obtained through Rosetta Commons. Filters relevant for this work under https://www.rosettacommons.org/docs/latest/scripting_documentation/RosettaScripts/Filters/Filters-RosettaScripts. For executing the python dependent scripts, PyRosetta and the Anaconda2 package Python 2.7.16, default, Sep 24 2019 are required. Scripts, XML files and examples have been deposited on github [https://github.com/strauchlab/scaffold_design]. Scripts to fit protease sequencing counts are available under https://github.com/asford/protease_experimental_analysis.

## References

[1] Huang PS, Boyken SE, Baker D (2016). The coming of age of de novo protein design. Nature.

[2] Jaroszewski L (2009). Exploration of uncharted regions of the protein universe. PLoS Biol..

[3] Koga N (2012). Principles for designing ideal protein structures. Nature.

[4] Lin YR (2015). Control over overall shape and size in de novo designed proteins. Proc. Natl Acad. Sci. USA.

[5] Marcos E (2018). De novo design of a non-local beta-sheet protein with high stability and accuracy. Nat. Struct. Mol. Biol..

[6] Pan X (2020). Expanding the space of protein geometries by computational design of de novo fold families. Science.

[7] Kosuri S, Church GM (2014). Large-scale de novo DNA synthesis: technologies and applications. Nat. Methods.

[8] Klein JC (2016). Multiplex pairwise assembly of array-derived DNA oligonucleotides. Nucleic Acids Res..

[9] Rocklin GJ (2017). Global analysis of protein folding using massively parallel design, synthesis, and testing. Science.

[10] Anishchenko I (2021). De novo protein design by deep network hallucination. Nature.

[11] Wintjens RT, Rooman MJ, Wodak SJ (1996). Automatic classification and analysis of alpha alpha-turn motifs in proteins. J. Mol. Biol..

[12] Jacobs TM (2016). Design of structurally distinct proteins using strategies inspired by evolution. Science.

[13] Simons, K. T., Bonneau, R., Ruczinski, I., Baker, D. Ab initio protein structure prediction of CASP III targets using ROSETTA. *Proteins* **Suppl 3**, 171–176 (1999). 10.1002/(sici)1097-0134(1999)37:3+<171::aid-prot21>3.3.co;2-q 10526365

[14] Kuhlman B (2003). Design of a novel globular protein fold with atomic-level accuracy. Science.

[15] Fallas JA (2017). Computational design of self-assembling cyclic protein homo-oligomers. Nat. Chem..

[16] Drew ED, Janes RW (2020). PDBMD2CD: providing predicted protein circular dichroism spectra from multiple molecular dynamics-generated protein structures. Nucleic Acids Res..

[17] Mirdita M (2022). ColabFold: making protein folding accessible to all. Nat. Methods.

[18] Huang PS (2014). High thermodynamic stability of parametrically designed helical bundles. Science.

[19] Brunette TJ (2015). Exploring the repeat protein universe through computational protein design. Nature.

[20] Pedregosa F (2011). Scikit-learn: machine learning in python. J. Mach. Learn. Res..

[21] Fleishman SJ (2011). RosettaScripts: a scripting language interface to the Rosetta macromolecular modeling suite. PloS one.

[22] Cao L (2020). De novo design of picomolar SARS-CoV-2 miniprotein inhibitors. Science.

[23] Cao L (2022). Design of protein-binding proteins from the target structure alone. Nature.

[24] Hoover DM, Lubkowski J (2002). DNAWorks: an automated method for designing oligonucleotides for PCR-based gene synthesis. Nucleic Acids Res..

[25] Benatuil L, Perez JM, Belk J, Hsieh CM (2010). An improved yeast transformation method for the generation of very large human antibody libraries. Protein Eng., Des. selection: PEDS.

[26] Whitehead TA (2012). Optimization of affinity, specificity and function of designed influenza inhibitors using deep sequencing. Nat. Biotechnol..

[27] Chao G (2006). Isolating and engineering human antibodies using yeast surface display. Nat. Protoc..

[28] Zhang J, Kobert K, Flouri T, Stamatakis A (2014). PEAR: a fast and accurate Illumina Paired-End reAd mergeR. Bioinformatics.

[29] Studier FW (2005). Protein production by auto-induction in high density shaking cultures. Protein Expr. Purif..

[30] Wishart DS, Bigam CG, Holm A, Hodges RS, Sykes BD (1995). 1H, 13C and 15N random coil NMR chemical shifts of the common amino acids. I. Investigations of nearest-neighbor effects. J. Biomol. NMR.

[31] Guntert P, Buchner L (2015). Combined automated NOE assignment and structure calculation with CYANA. J. Biomol. NMR.

[32] Shen Y, Bax A (2013). Protein backbone and sidechain torsion angles predicted from NMR chemical shifts using artificial neural networks. J. Biomol. NMR.

[33] Brunger AT (2007). Version 1.2 of the crystallography and NMR system. Nat. Protoc..

[34] Brunger AT (1998). Crystallography & NMR system: A new software suite for macromolecular structure determination. Acta Crystallogr D. Biol. Crystallogr.

[35] Koradi R, Billeter M, Wuthrich K (1996). MOLMOL: a program for display and analysis of macromolecular structures. J. Mol. Graph.

[36] Laskowski RA, Rullmannn JA, MacArthur MW, Kaptein R, Thornton JM (1996). AQUA and PROCHECK-NMR: programs for checking the quality of protein structures solved by NMR. J. Biomol. NMR.

[37] Hutchinson EG, Thornton JM (1996). PROMOTIF–a program to identify and analyze structural motifs in proteins. Protein Sci.: a Publ. Protein Soc..

[38] Williams CJ (2018). MolProbity: More and better reference data for improved all-atom structure validation. Protein Sci.: a Publ. Protein Soc..

