# Supplementary material for: Sampling of structure and sequence space of small protein folds
Source: Nat Commun. 2022 Nov 22;13:7151. doi: 10.1038/s41467-022-34937-8 (PMC9684540; doi:10.1038/s41467-022-34937-8)
Supplement: Supplementary file 1 — Supplementary Information [file 41467_2022_34937_MOESM1_ESM.pdf]

Supplementary material for

Sampling of Structure and Sequence Space of Small Protein Folds

Thomas Linsky[1,2,#], Kyle Noble[3,#], Autumn R Tobin[3,#], Rachel Crow[4,#], Lauren Carter[2], Jeffrey L Urbauer [5], David Baker[1,2,6], Eva-Maria Strauch[3,7]*

[1]*Department of Biochemistry, University of Washington, Seattle, WA 98195, USA.*
[2]*Institute for Protein Design, University of Washington, Seattle, WA 98195, USA.*
[3]*Department of Pharmaceutical and Biomedical Sciences, University of Georgia, Athens, GA 30602, USA.*
[4]*Department of Microbiology, University of Washington, Seattle, WA 98195, USA.*
[5]*Department of Chemistry, University of Georgia, Athens, GA 30602, USA.*
[6] *Howard Hughes Medical Institute, University of Washington, Seattle, WA 98195, USA*
[7]*Institute of Bioinformatics, University of Georgia, Athens, GA 30602, USA.*

#Contributed equally
*Corresponding author, estrauch@uga.edu

Supplementary Information

Supplementary Figures 1-23

Supplementary Tables 1-2

Supplementary Methods

Supplementary figures

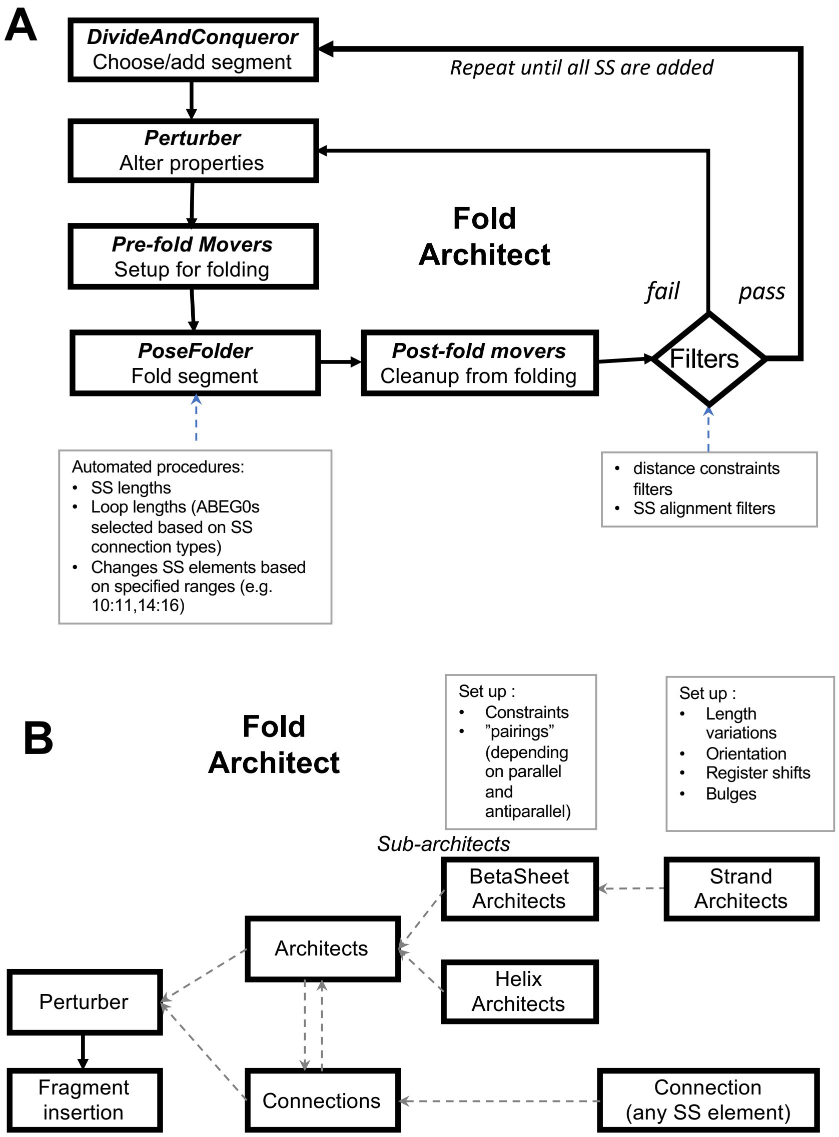

Supplementary Figure 1. Workflow for backbone design. (A) succession of classes that are part of the FoldArchitect and its input description. After deciding on a specific segment (two secondary structure elements with their connecting loop), the perturber selects the properties of the segment, including specific lengths and loop ABEGO variations. After folding through the PoseFolder, poses are evaluated by the filters, which can check distances, secondary structure pairing, or hydrogen bonding. (B) Break down of the subclasses that describe the individual secondary structures and their dependencies.

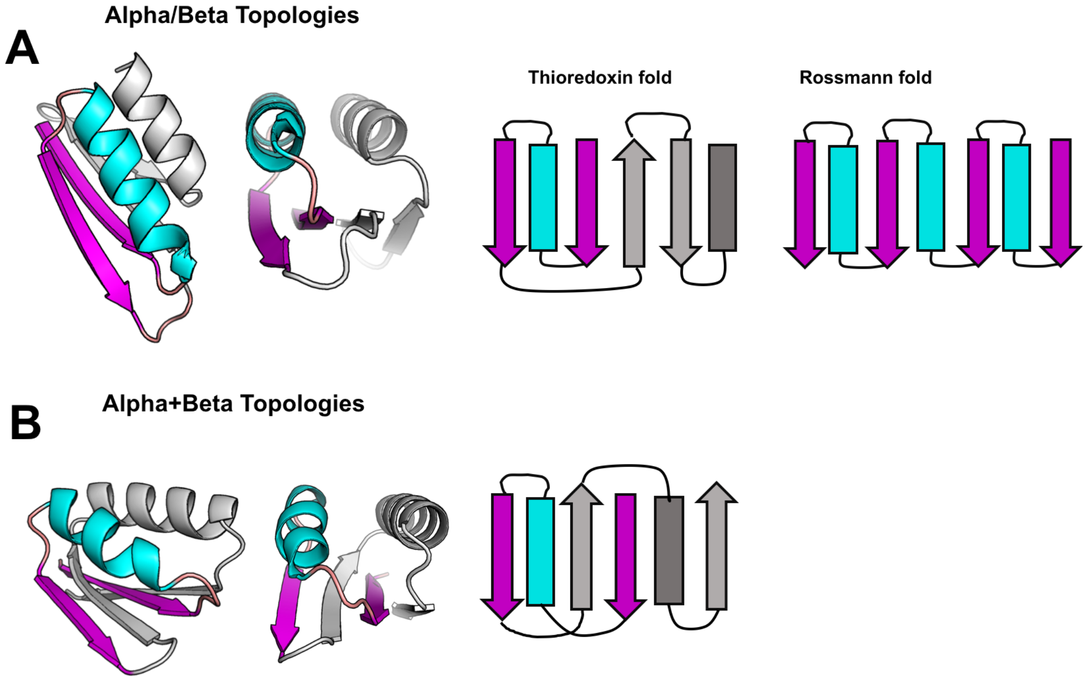

Supplementary Figure 2. **Differences between α/β and α+β folds**. (A) The α/β family contains the often-repeated units of the classical β-α-β motif, such that there is a repetition of this arrangement (e.g. as observed in the Rossman fold. The beta strands are parallel, and hydrogen-bonded to each other. When multiple β-α-β a linked, the alpha helices are all parallel to each other, and are antiparallel to the strands. Thioredoxin is the smallest representative of this family as it has only one β-α-β motif. (B) The α+β domain family comprises folds that include significant alpha and beta secondary structural elements, but their elements are intermixed. The β-strands are therefore mostly *antiparallel*. Examples include ferredoxin fold, ribonuclease A, and the SH2 domain.

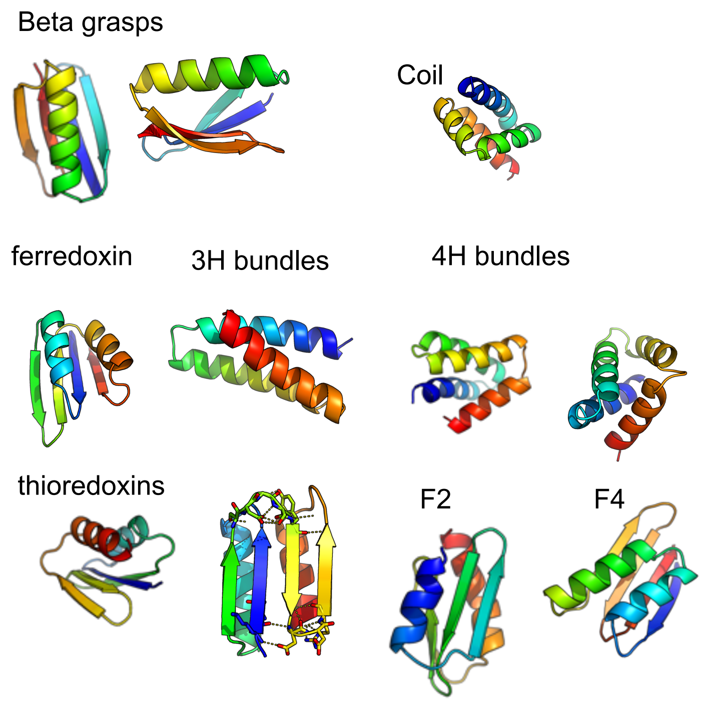

Supplementary Figure 3. Overview of sampled topologies that can be encoded by 64 residues or less.

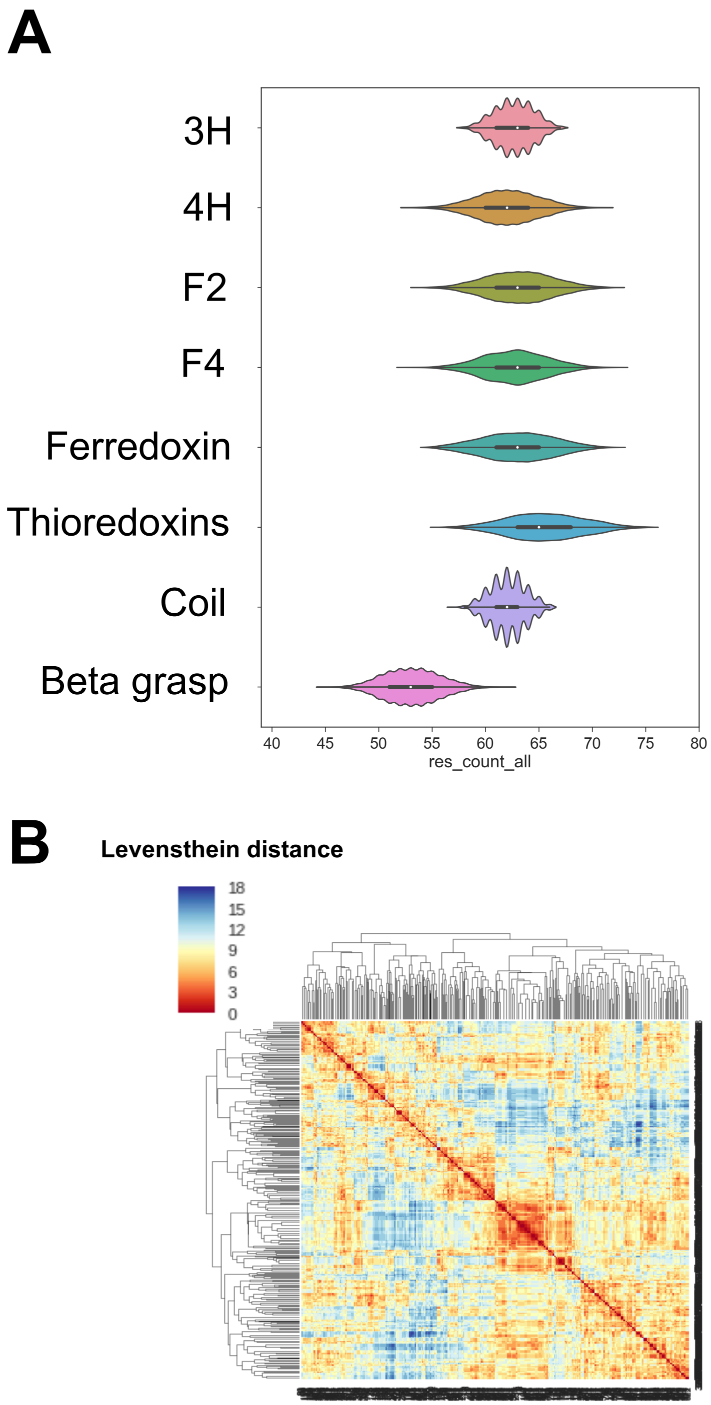

Supplementary Figure 4. Sampled diversity for backbone generation. (A) Length distribution of backbones generated using the FoldArchitect. Only backbones that had less than 65 amino acids were subjected to sequence design and subsequentially tested using the protease-based yeast surface display screen. (B) Comparison of 3,500 backbone designs for ferredoxins show that the ABEGO sequences of generated designs are highly diverse. Comparison of ABEGO sequences were computed using Levensthein distances which are reflected in their color. ABEGO sequences were clustered to demonstrate variations.

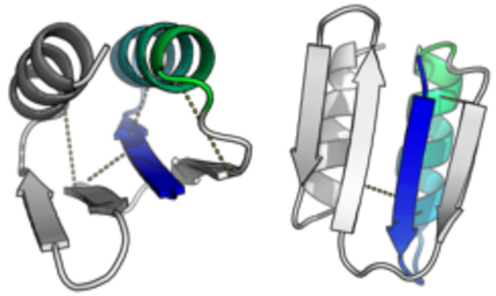

Supplementary Figure 5. Starting point and distance restraints used for thioredoxin folds. For each fold, two starting segments are selected to be built first. For most folds, the middle segments were used, but for the thioredoxin, multiple starting points for the assembly were tested. Starting with the first strand and helix resulted in the most decoys passing all filters and was thus used for the backbone generation protocol. Loose harmonic distance restraints were defined for each element using Rosetta AtomPairConstraints to encourage the intended tertiary structure (dotted yellow lines).

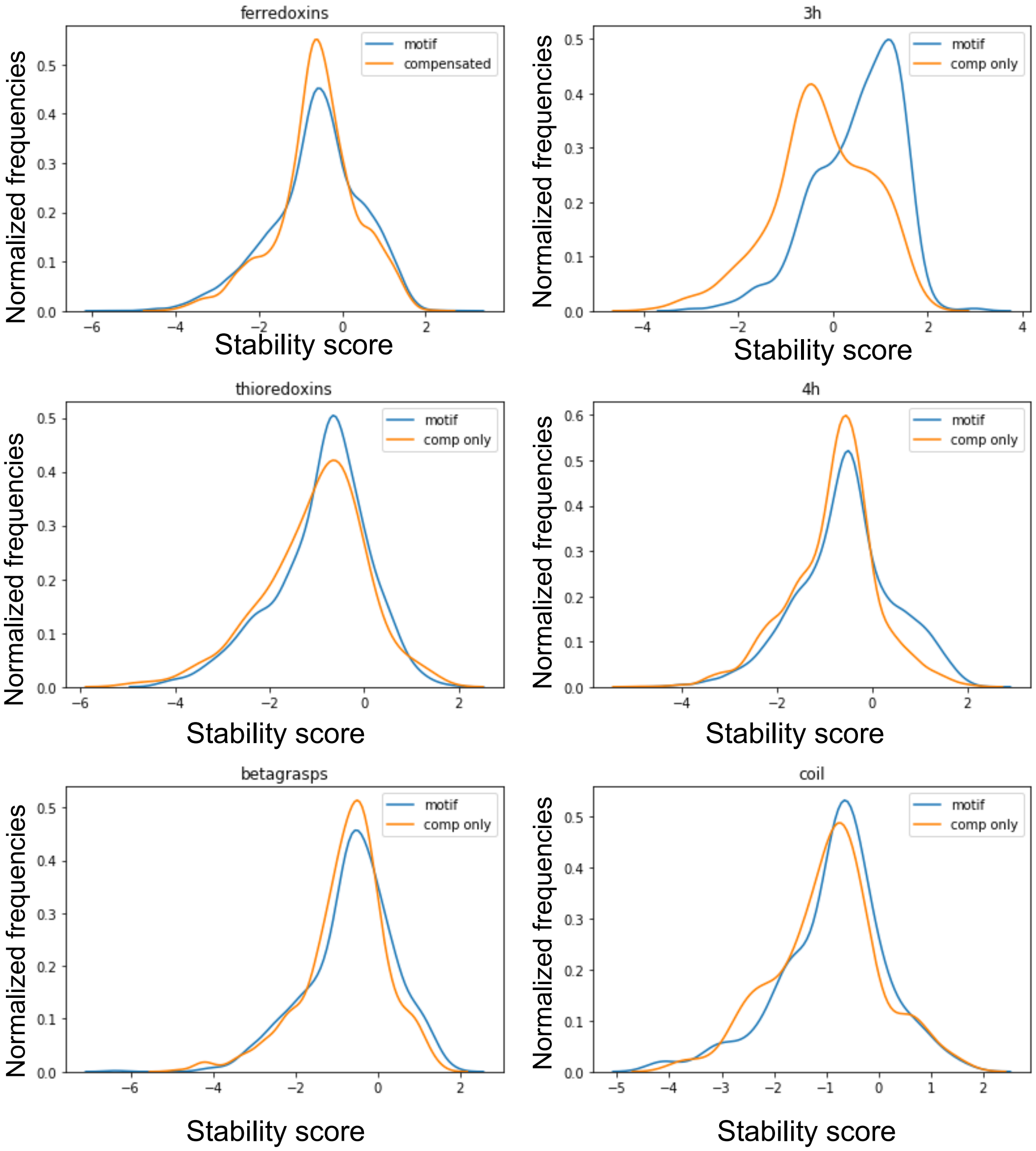

Supplementary Figure 6. Distributions of stability scores for different folds. Two sequence design protocols were applied to the generated backbones: "motif" and "simple". "Motif" indicates the use of pair motifs during the FastDesign protocol whereas "simple" is using the FastDesign protocol without pair motifs. F2 and F4 were designed only with motifs and are therefore not illustrated here. We compared the stability scores for each design and separated two populations based on the design protocol used for sequence design.

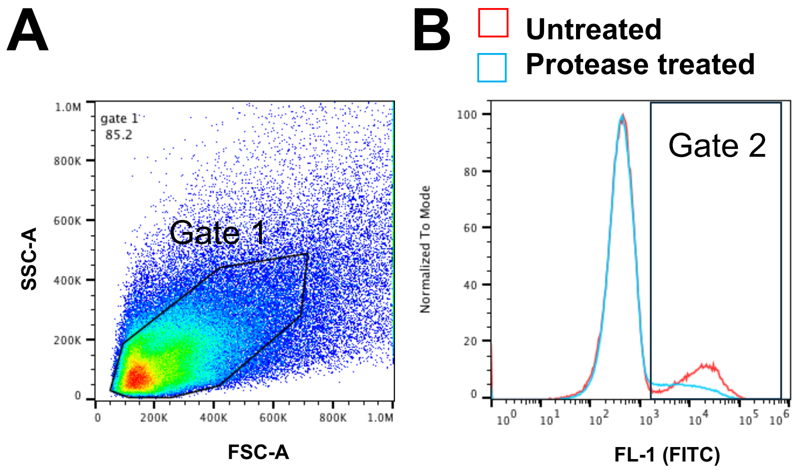

Supplementary Figure 7. Gating strategy for fluorescence activated cell sorting (FACS). (A) Yeast cells were selected based on their forward (FSC-A) and sideward scattering intensities. (B) Before sorting protease digested yeast cells displaying the designs, the control labeled with anti-cMyc antibody conjugated to FITC was used to set gate 2, which allows selection of displaying cells

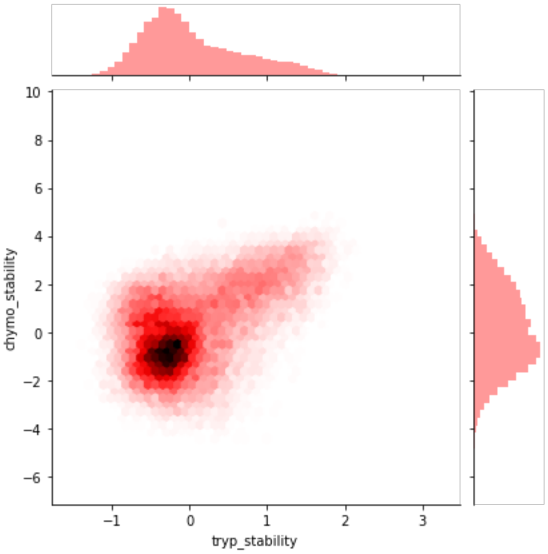

Supplementary Figure 8. **Stability scores**. EC50-based stability scores for all data point with a high confidence fit for chymotrypsin (y-axis) and trypsin (x-axis) describing 31,180 sequences in the experimental dataset.

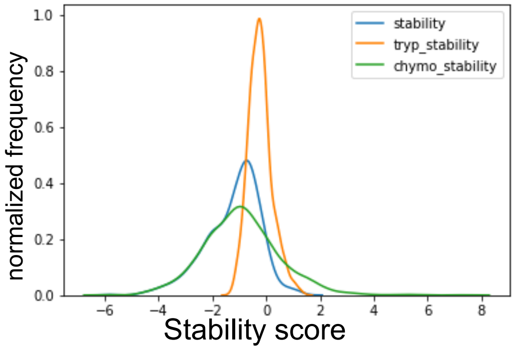

Supplementary Figure 9. **Stability scores for randomized sequences.** Trypsin- ("tryp_stability") and chymotrypsin-based ("chymo_stability") stability scores of 2,300 randomly scrambled sequences, which are assumed to be unstable, that were added as a control. A general stability score ("stability") was computed by taking the minimum of the trypsin and chymotrypsin stability scores. Based on the general stability values of the scrambled sequences, a threshold of 0.5 was selected to distinguish between stable and unstable designs.

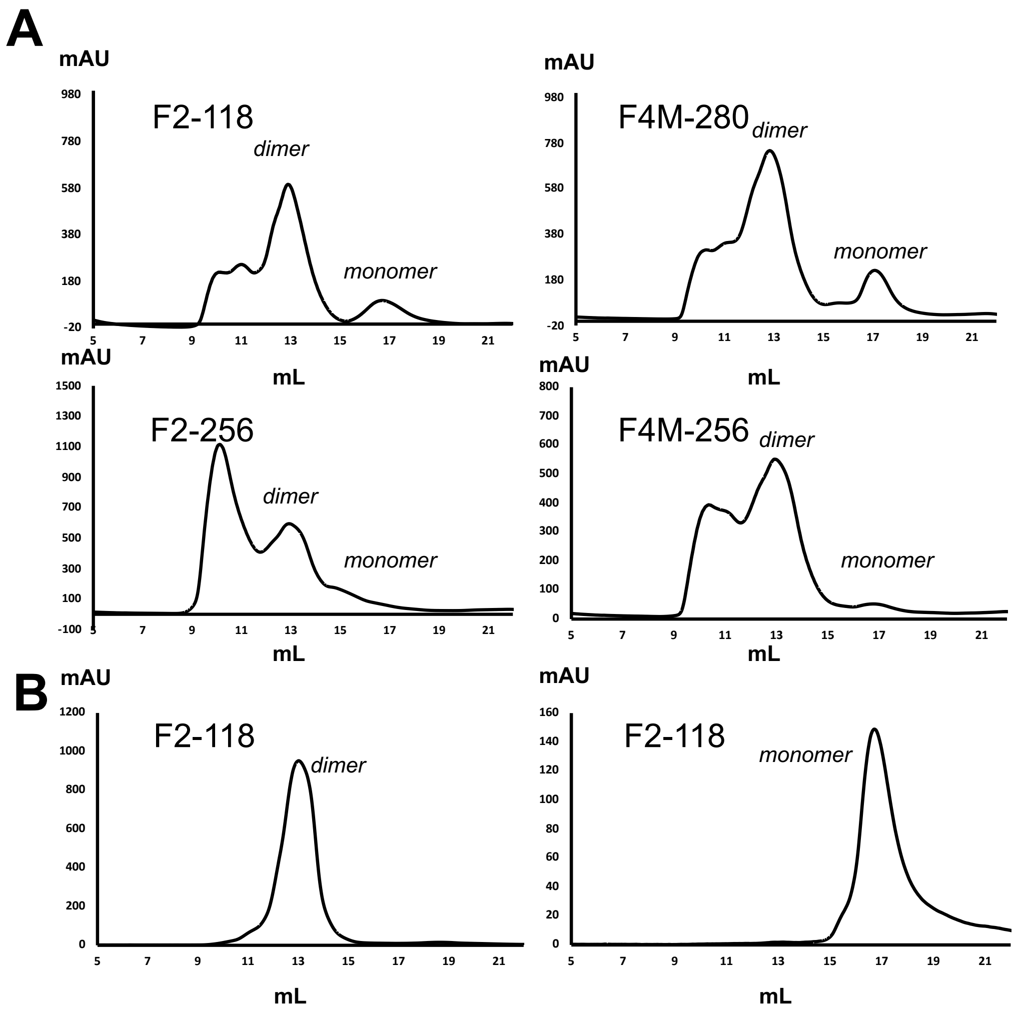

Supplementary Figure 10. Size exclusion chromatography of F2 and F4. (A) SEC traces after Ni-NTA purification. As several of these proteins have no tryptophan residues, we used 215 nm as wavelength to monitor elution from a chromatography run using a Superdex S75 (10/300). (B) Re-run of the dimer and monomer fraction of F2-118 after one-month storage at 4° C. There appears to be no equilibrium between the monomer and dimer fractions.

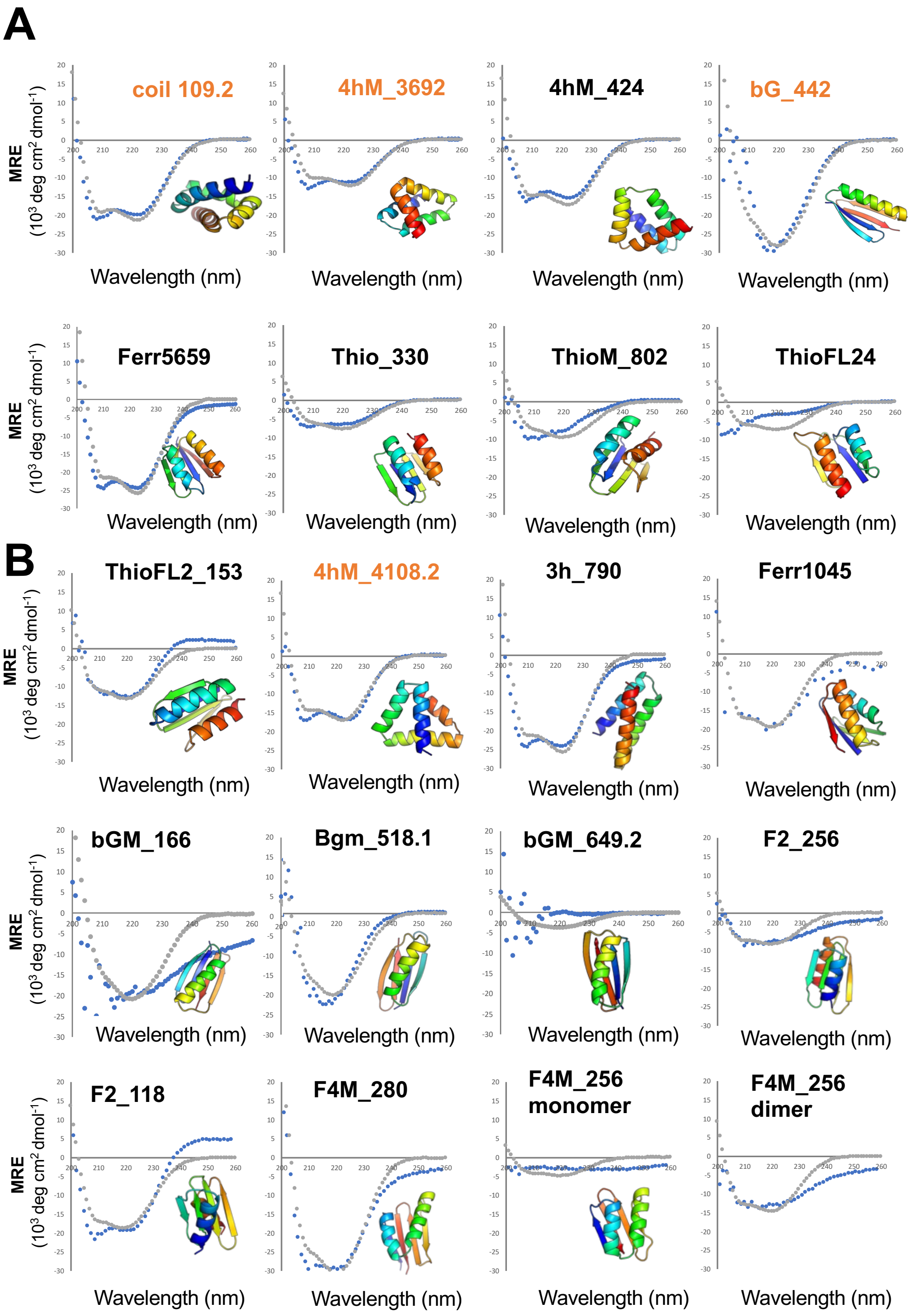

Supplementary Figure 11. Circular dichroism and models of proteins examined. (A) Experimentally determined CD spectra (blue), as described in Fig. 3, and predicted CD spectra (gray) calculated using the program PDBMC2CDD[1]. (B) Experimentally determined CD spectra and predictions for additional proteins identified as stable as part of the protease high throughput screen as summarized in Table S1. For measurements using the Olis CD spectrophotometer, 5 data points were averaged and plotted here. Ferr1045 was measured using the Aviv CD spectrophotometer with data points representing the average of 3 measurements. All measurements used a 1 second integration time. Unless noted, the monomeric fraction was examined. Proteins for which 1D proton spectra were obtained (Figure S11) are labeled in orange.

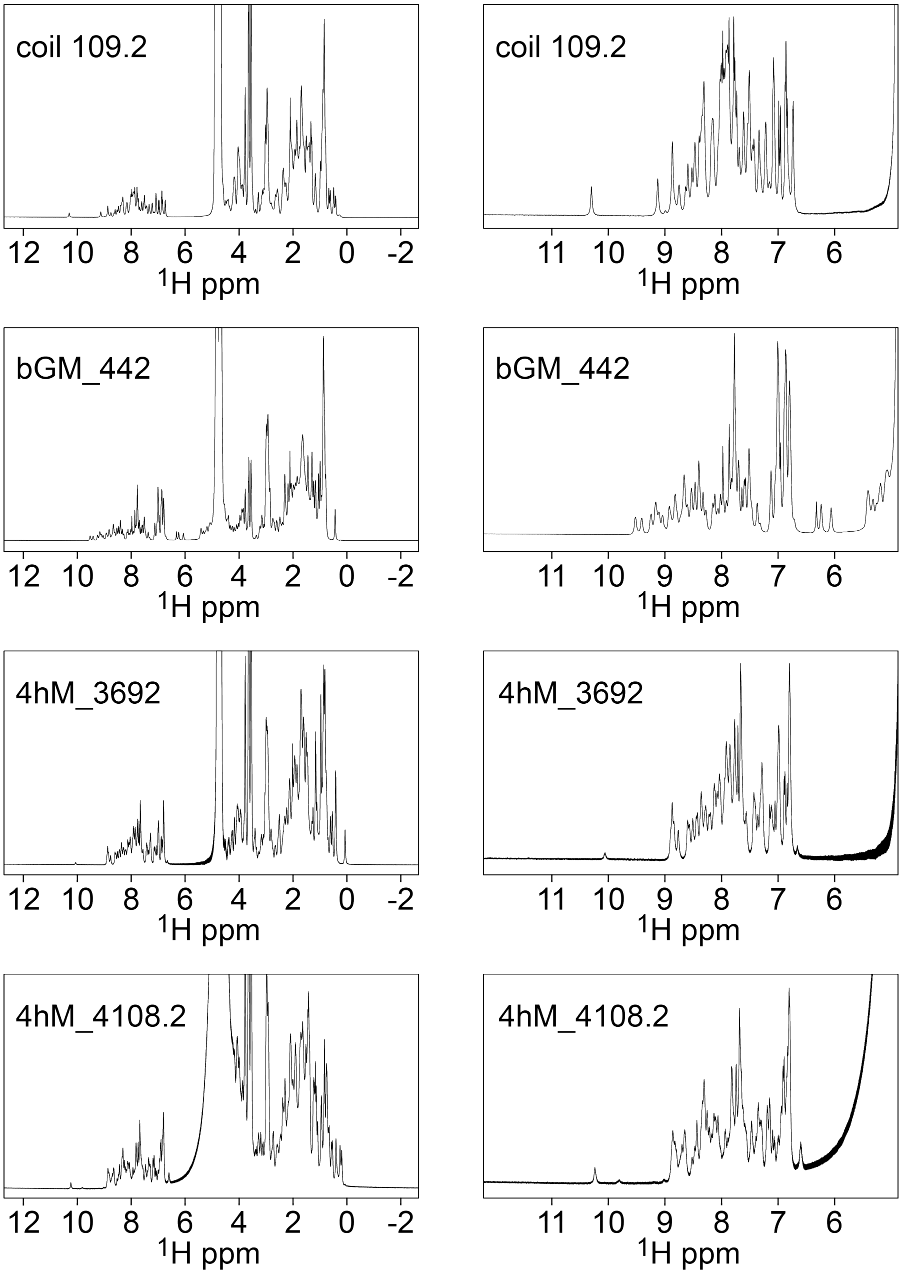

Supplementary Figure 12. 1D, ^{1}H NMR spectra of four of the designed proteins (coil 109.2, bGM_442, 4hM_3692, and 4h_4108.2). Shown are full spectra and the downfield regions (aromatic and amide hydrogens) for each. The large peaks at ~3.6 ppm are from residual glycerol. The water signal (4.76 ppm) appears particularly large in the spectrum of 4hM_4108.2 because the protein was more dilute.

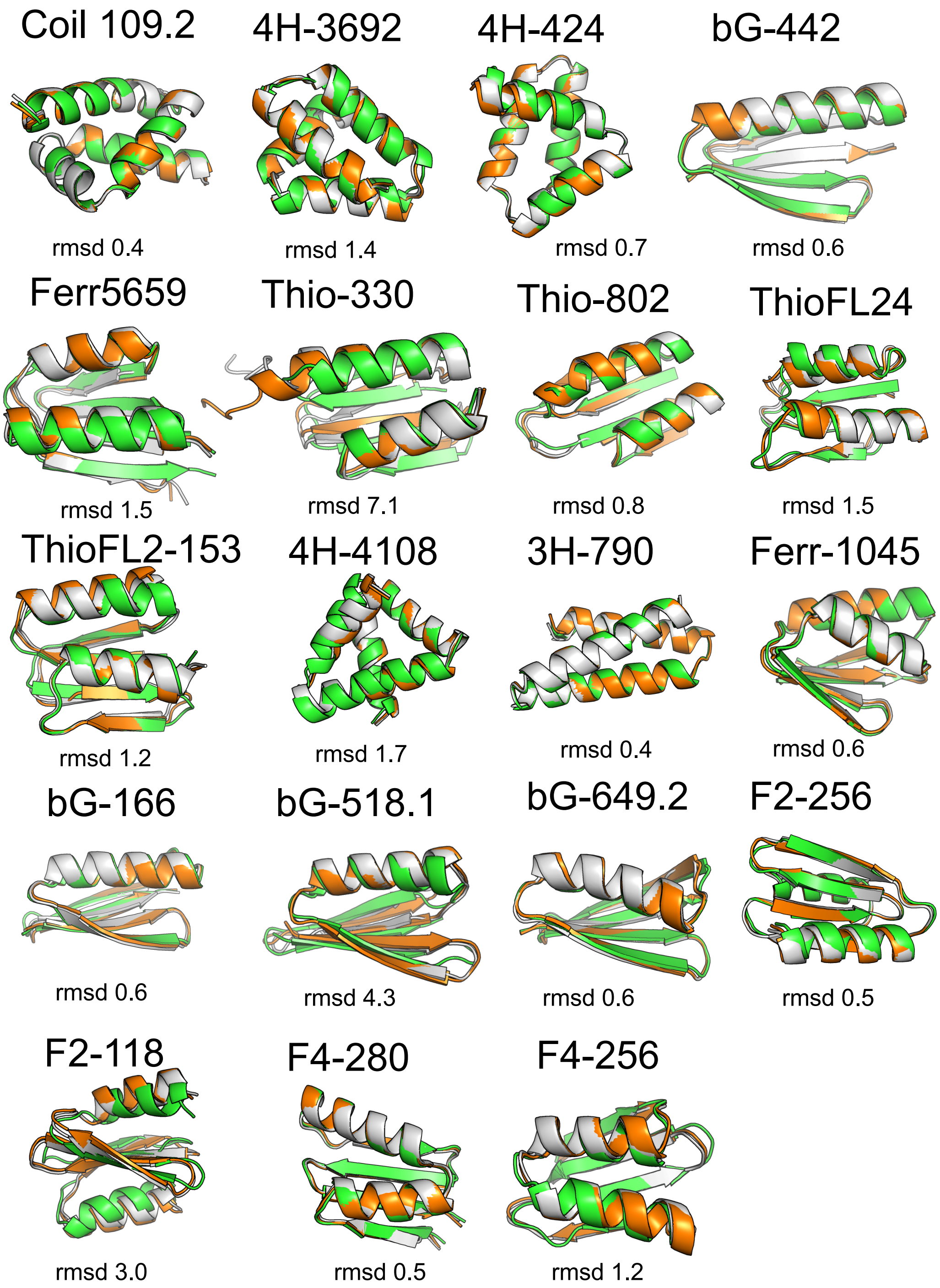

Supplementary Figure 13. Structure predictions of the characterized folds by AlphaFold2. Original design models (green) were predicted using the ColabFold[2] interface (without multiple sequence alignment and template). The top ranked prediction model (grey) was relaxed by a repacking and minimization step using Rosetta (orange) and the rmsd (reported below each design) of the relaxed prediction was compared to the original design model.

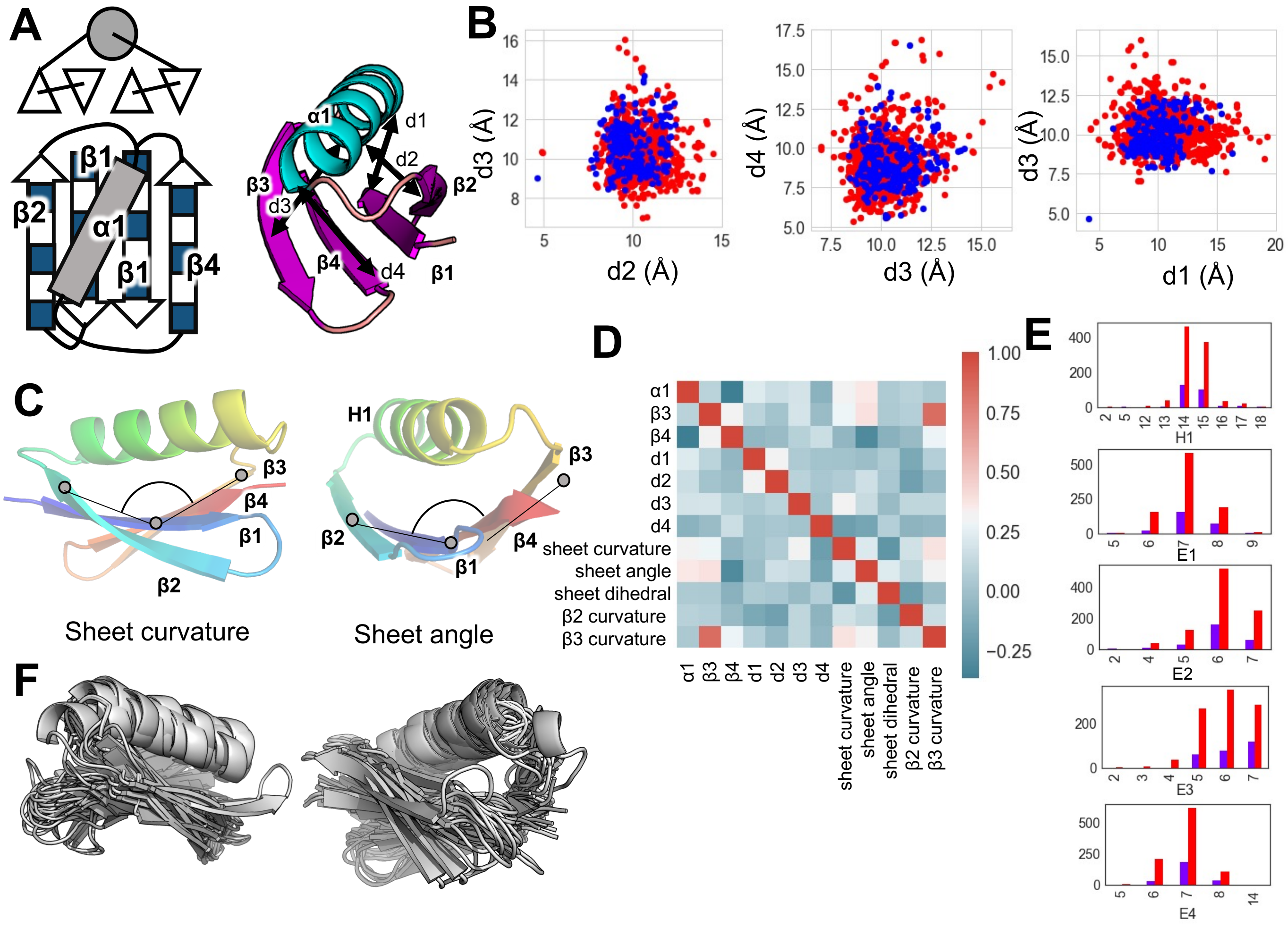

Supplementary Figure 14. Geometric analysis of beta-grasps. (A) Schematic of the beta-grasp fold and definition of distances between midpoints of strands and helix. (B) Plots of distances between strands and helix; blue represents stable scaffolds and red unstable. (C) Illustration defining sheet curvature and sheet angle. (D) Correlations of distances, specific secondary structure lengths and angles for stable folds. (E) Summary of lengths of strands and helix for stable (blue) and unstable (red) designs. (F) Superposition of 20 designed beta-strand folds to illustrate structural and shape diversity.

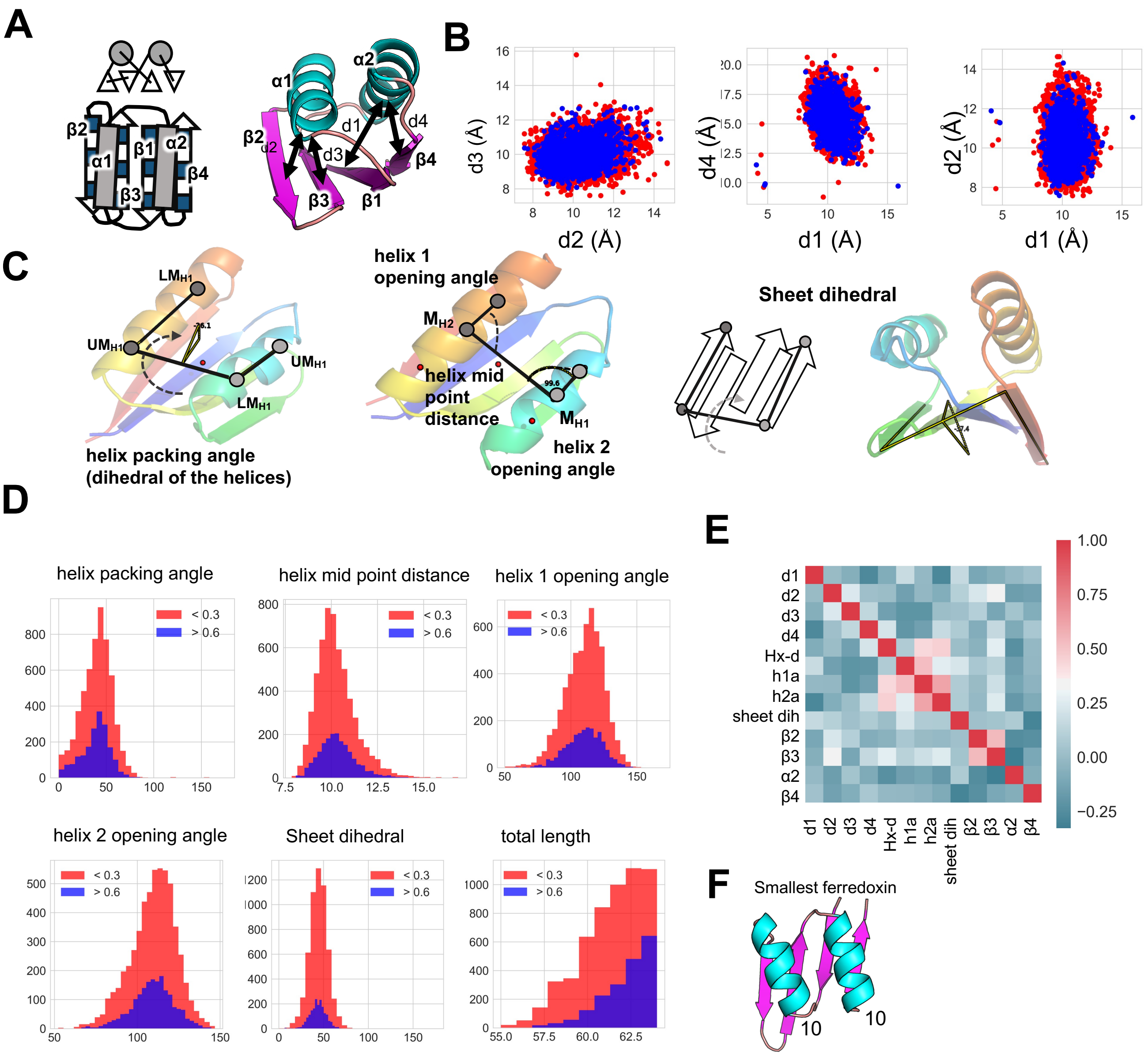

Supplementary Figure 15. Geometric analysis of designed and assayed ferredoxins. (A) Schematic of the ferredoxin fold and definition of distances between midpoints of strands and helices that were measured here. (B) Scatter plots of distances between strands and helices; blue represents stable scaffolds and red unstable. (C) Illustration defining helix distances, angles and sheet dihedrals. (D) Distributions of angles and distances for stable (blue) and unstable (red) designs. (E) Correlations of distances, specific secondary structure lengths (β 1-4, α2) and angles (h1a = helix 1 opening angle, h2a = helix 2 opening angle, sheet dih = sheet dihedral) for stable folds. (F) Model of the smallest, stable ferredoxin sampled; it is 55 residues long.

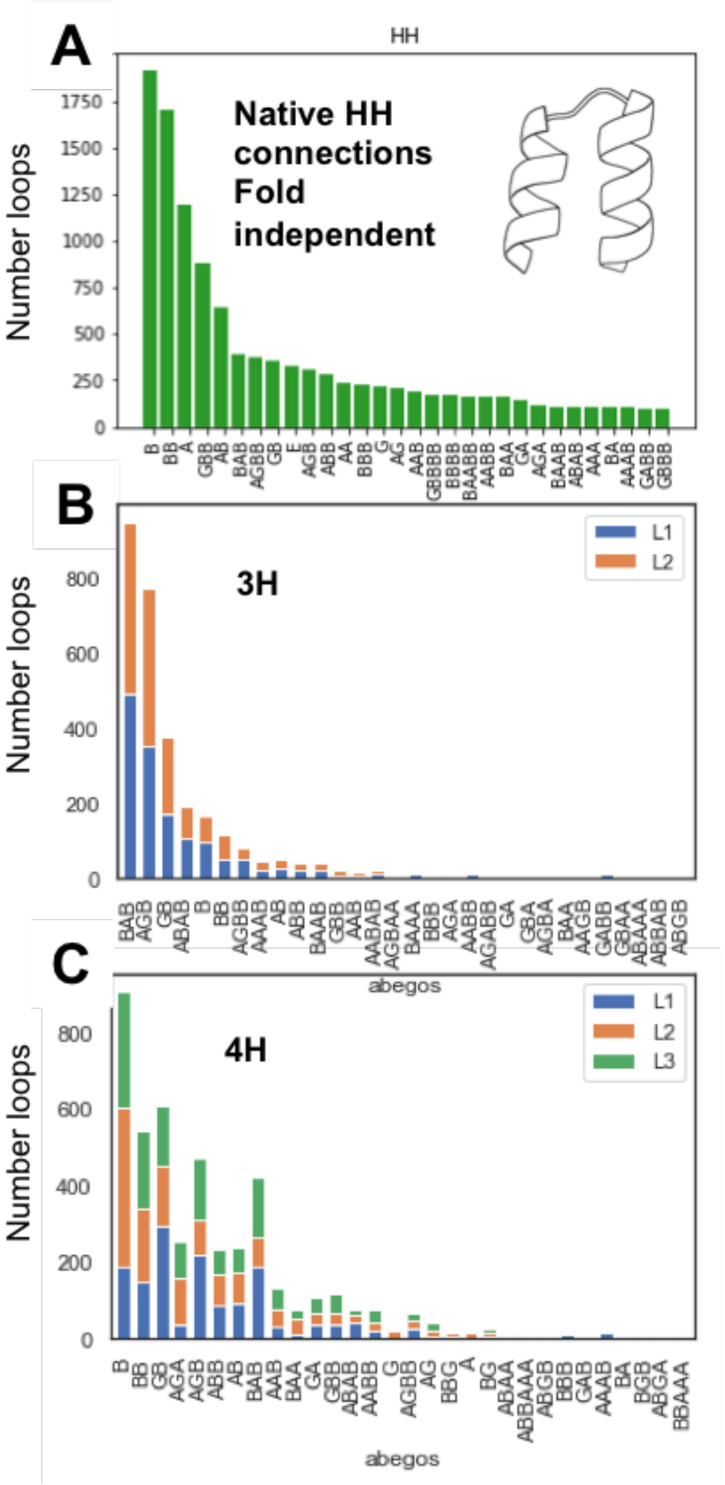

Supplementary Figure 16. Rules for connecting helical elements. (A) Distributions of loop connections found in 7000 high resolution crystal structures and (B) found in the stable 3-helical (3H) or (C) 4-helical bundles (4H) of this work.

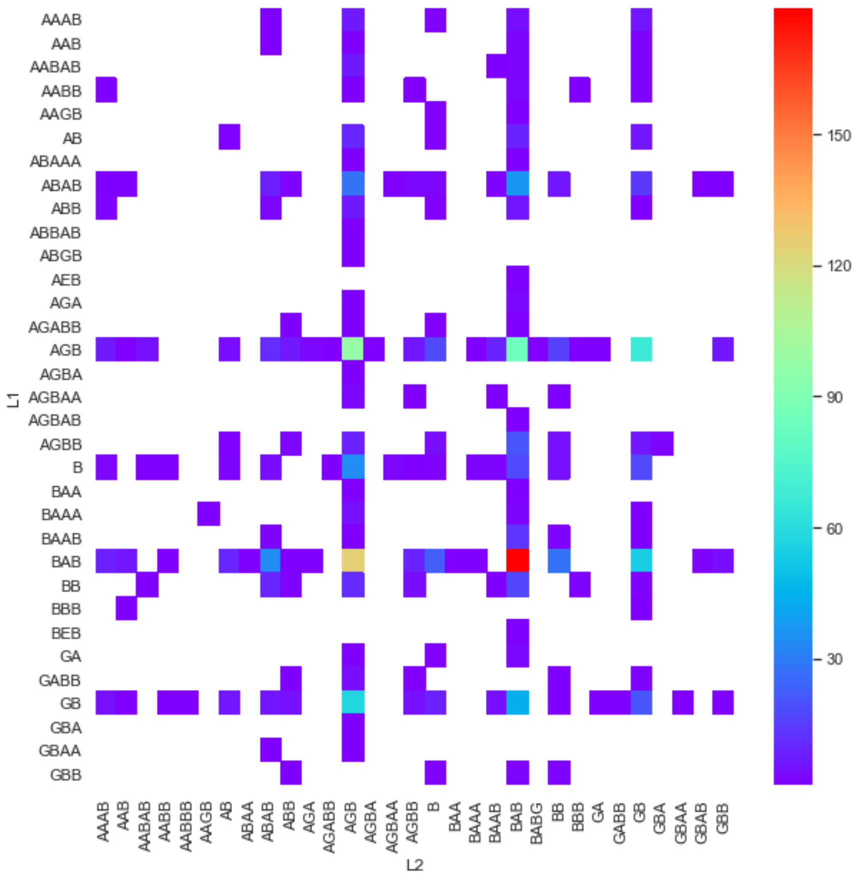

Supplementary Figure 17. Loop angles described as ABEGO letters for stable 3H bundles. L1 as y-axis and L2 as x-axis. Color describes how often it was seen (red=present in in more designs, blue=present in fewer designs).

A

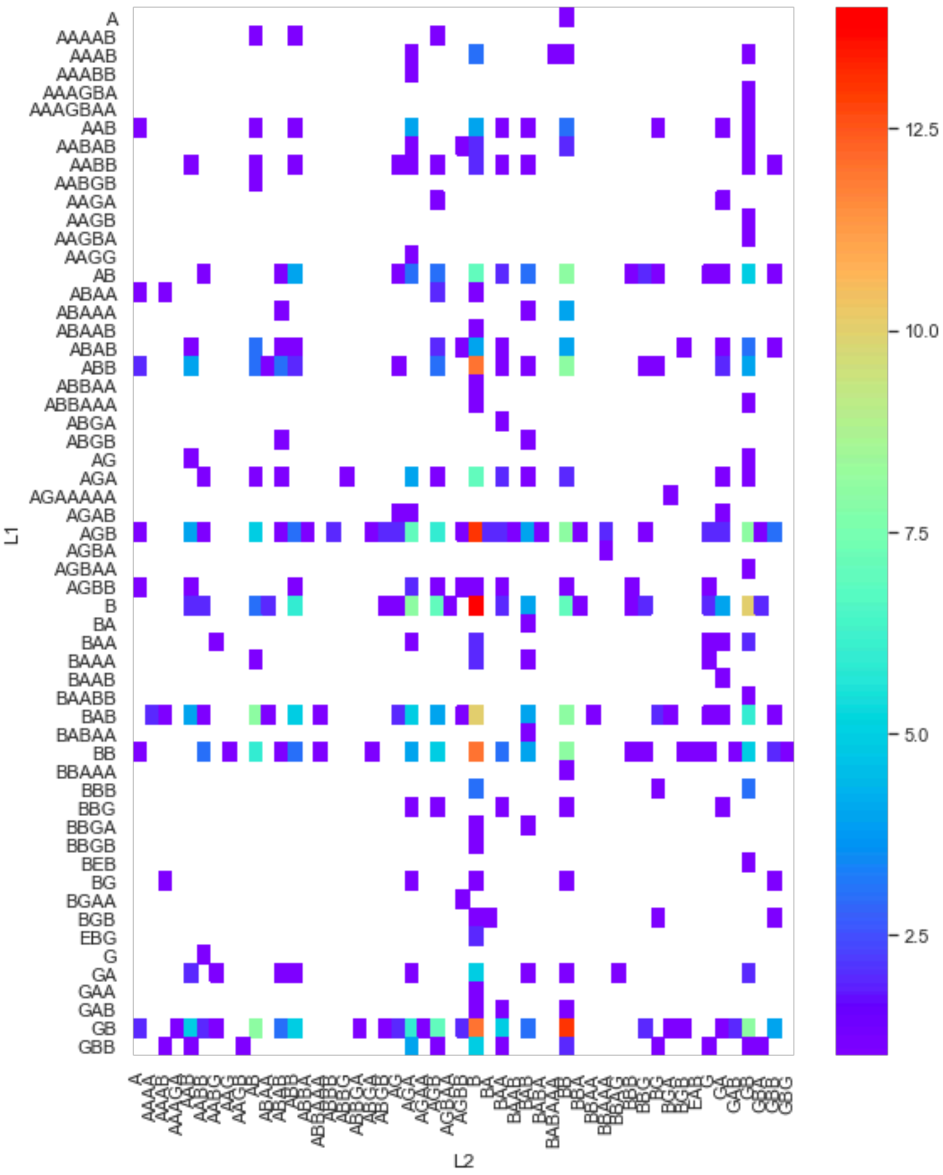
L1
L2
A
AAAAB
AAAB
AAABB
AAAGBA
AAAGBAA
AAB
AABAB
AABB
AABGB
AAGA
AAGB
AAGBA
AAGG
AB
ABAA
ABAAA
ABAAB
ABAB
ABB
ABBAA
ABBAAA
ABGA
ABGB
AG
AGA
AGAAAAA
AGAB
AGB
AGBA
AGBAA
AGBB
B
BA
BAA
BAAA
BAAB
BAABB
BAB
BABAA
BB
BBAAA
BBB
BBG
BBGA
BBGB
BEB
BG
BGAA
BGB
EBG
G
GA
GAA
GAB
GB
GBB
12.5
10.0
7.5
5.0
2.5

B

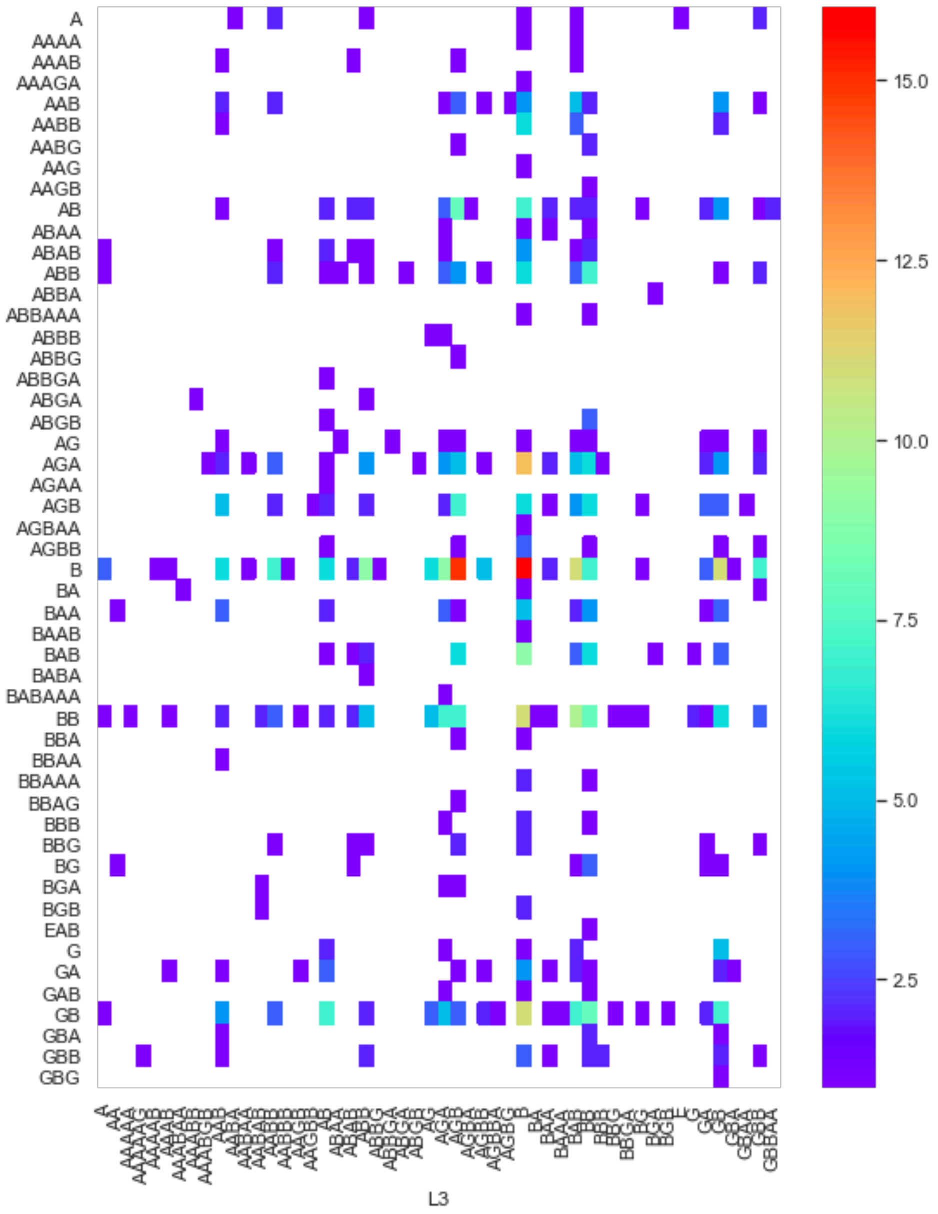

Supplementary Figure 18. Loop angles described as ABEGO letters for stable 4H bundles. (A) L1 as y-axis and L2 as x-axis. (B) L2 as y-axis and L3 as x-axis. Color describes how often it was seen. (red=present in in more designs, blue=present in fewer designs)

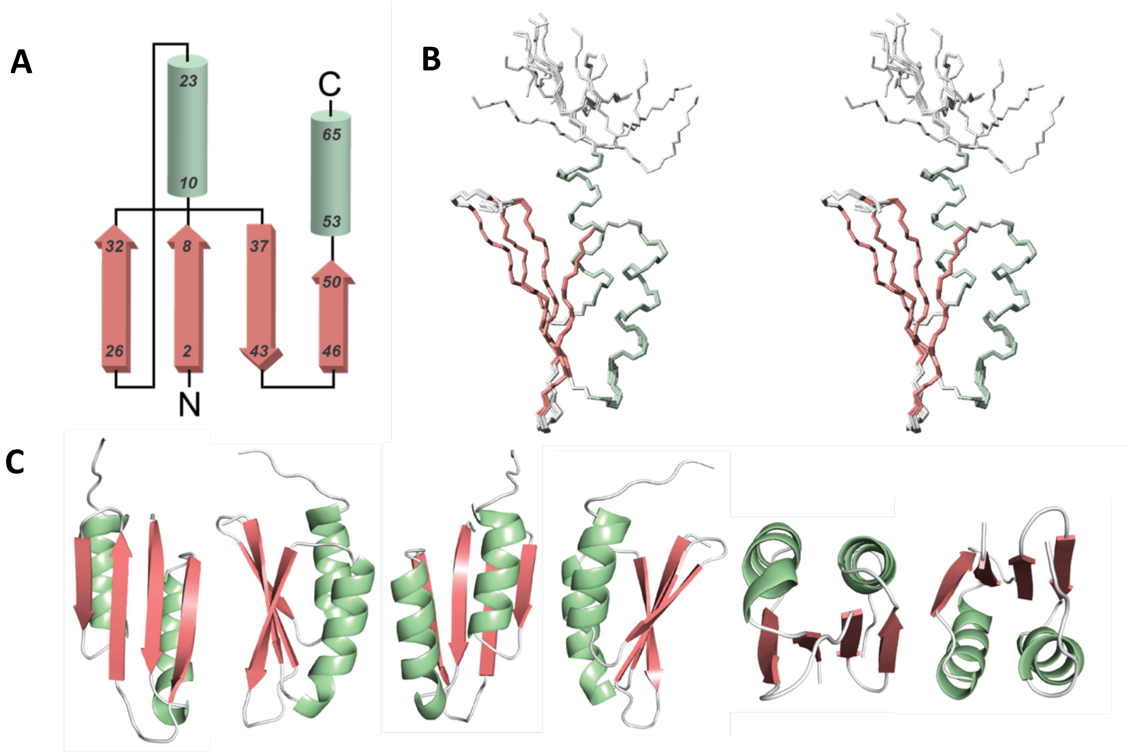

Supplementary Figure 19. Thio_802 structure and topology. (A) Topology 'wiring diagram' of the solution structure of the Thio_802 protein determined using NMR spectroscopy (no register information is implied). (B) Cross-eyed stereo view of the 20-structure ensemble (refined structures with the lowest overall energies) superimposed on the mean structure. (C) Various views of the ribbon diagram of the lowest energy structure of the ensemble.

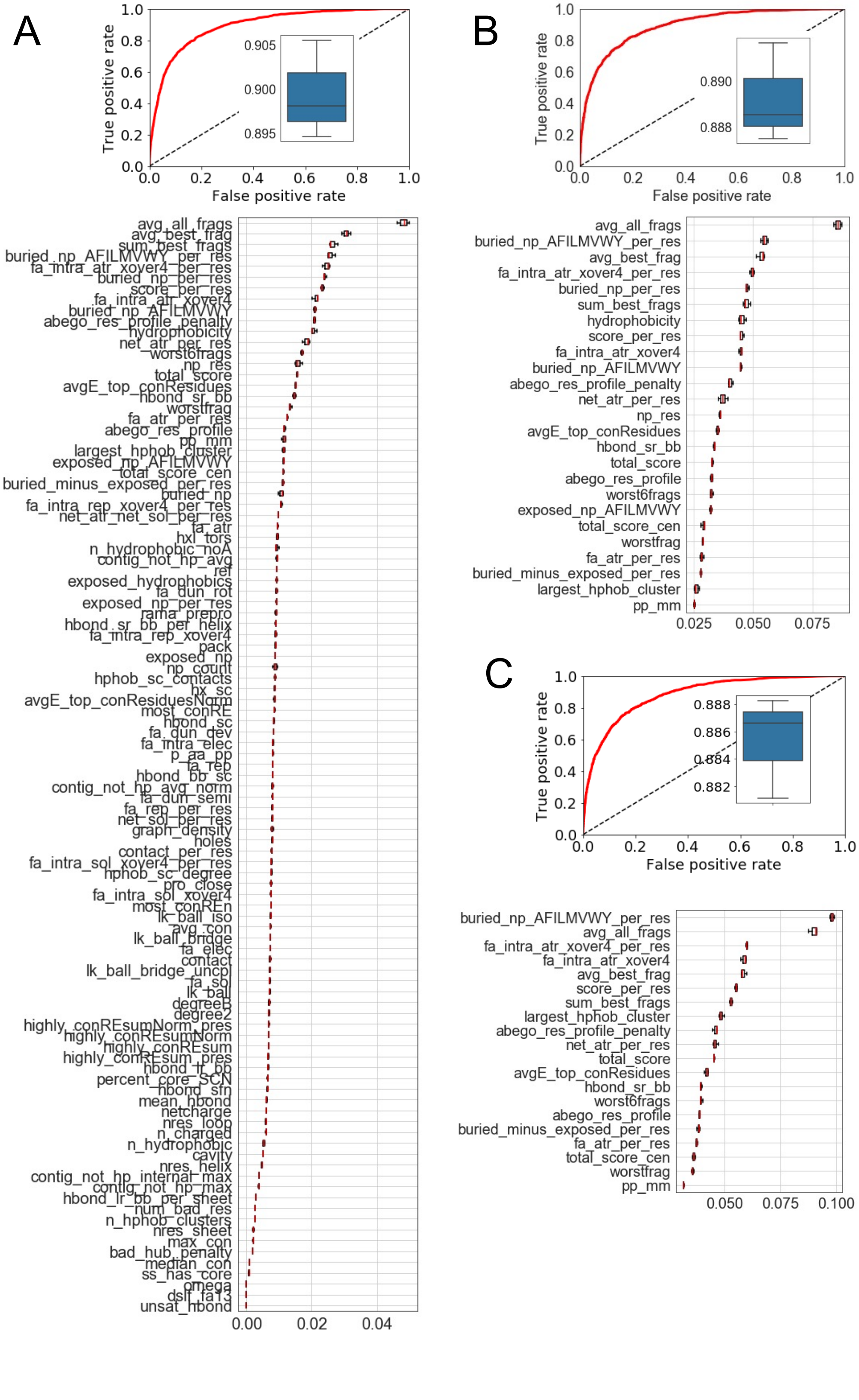
A
B
C
True positive rate
False positive rate
0.905
0.900
0.895
0.890
0.888
0.886
0.884
0.882
avg_all_frags
avg_best_frag
sum_best_frags
buried_np_AFILMVWY_per_res
fa_intra_atr_xover4_per_res
buried_np_per_res
score_per_res
fa_intra_atr_xover4
buried_np_AFILMVWY
abego_res_profile_penalty
hydrophobicity
net_atr_per_res
worst6frags
np_res
total_score
avgE_top_conResidues
hbond_sr_bb
worstfrag
fa_atr_per_res
abego_res_profile
pp_mm
largest_hphob_cluster
exposed_np_AFILMVWY
total_score_cen
buried_minus_exposed_per_res
buried_np
fa_intra_rep_xover4_per_res
net_atr_net_sol_per_res
fa_atr
hxl_tors
n_hydrophobic_noA
contig_not_hp_avg
ref
exposed_hydrophobics
fa_dun_rot
exposed_np_per_res
rama_prepro
hbond_sr_bb_per_helix
fa_intra_rep_xover4
pack
exposed_np
np_count
hphob_sc_contacts
hx_sc
avgE_top_conResiduesNorm
most_conRE
hbond_sc
fa_dun_dev
fa_intra_elec
p_aa_pp
fa_rep
hbond_bb_sc
contig_not_hp_avg_norm
fa_dun_semi
fa_rep_per_res
net_sol_per_res
graph_density
holes
contact_per_res
fa_intra_sol_xover4_per_res
hphob_sc_degree
pro_close
fa_intra_sol_xover4
most_conREn
lk_ball_iso
avg_con
lk_ball_bridge
fa_elec
contact
lk_ball_bridge_uncpl
fa_sol
lk_ball
degreeB
degree2
highly_conREsumNorm_pres
highly_conREsumNorm
highly_conREsum
highly_conREsum_pres
hbond_lr_bb
percent_core_SCN
hbond_sfn
mean_hbond
netcharge
nres_loop
n_charged
n_hydrophobic
cavity
nres_helix
contig_not_hp_internal_max
contig_not_hp_max
hbond_lr_bb_per_sheet
num_bad_res
n_hphob_clusters
nres_sheet
max_con
bad_hub_penalty
median_con
ss_has_core
omega
dslf_fa13
unsat_hbond
0.00
0.02
0.04
worst6frags
0.025
0.050
0.075
0.100

Supplementary Figure 20: **Features of prediction models.** (A) Receiver operating characteristic curves after taking 101 scoring features in account; we achieved an area under the curve (AUC) of about 0.9 for all designs, using 3-fold cross-validations with 20% of the data. (B) Taking the top 25 determining features to repeat training and prediction; the AUC is getting lower and feature importance changes, likely due to correlated terms. (C) After taking out several correlated features, training and prediction was done with 20 features. However, predictions did not improve. Thereby the complete set of features is most descriptive for the stability of these *de novo* designed proteins. This graph represents the minimum, maximum, median (red line), first quartile and third quartile in the data set.

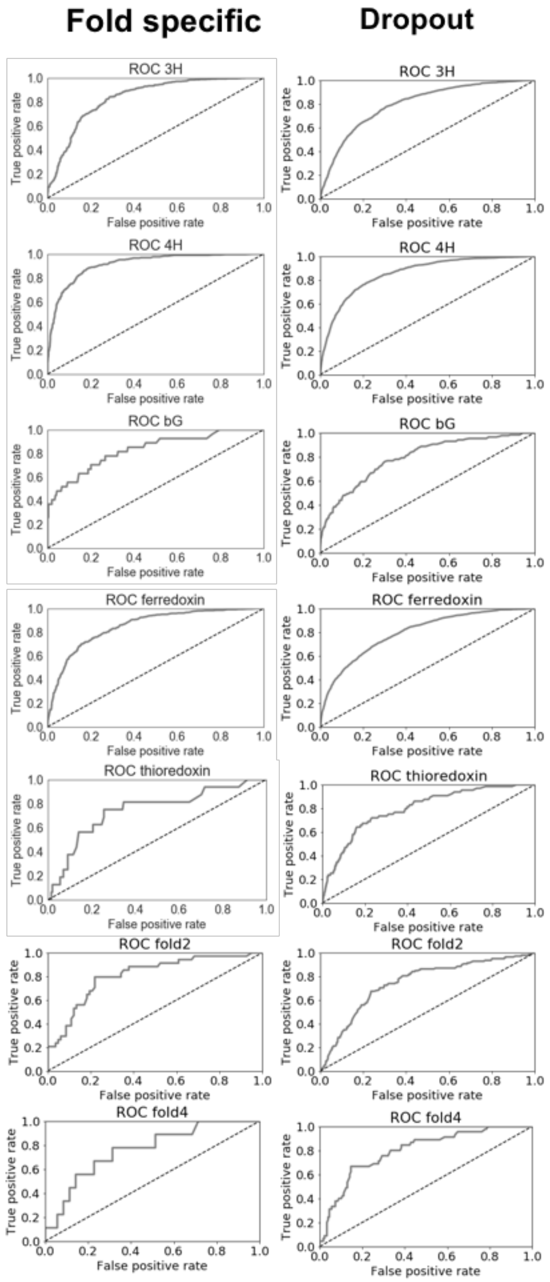

Supplementary Figure 21. Receiver operating characteristic curves (ROC) for stability prediction. Predictions of individual folds or using a dropout data set in which the fold to be predicted has not been used in training. The first scenario uses data from a fold, takes 80% of the stability data for the given fold for training and predicts false positives and true positive of the remaining 20%. For the "dropout" category, all protein sequences and data points are taken out of the complete set. The model is trained on all other folds and false and true positives for the specified fold are then predicted. Predictions for F2 and F4 and to some extent thioredoxin are noisy as the numbers are low. F2 has about 500, F4 has 222, and thioredoxin has about 1000 representatives.

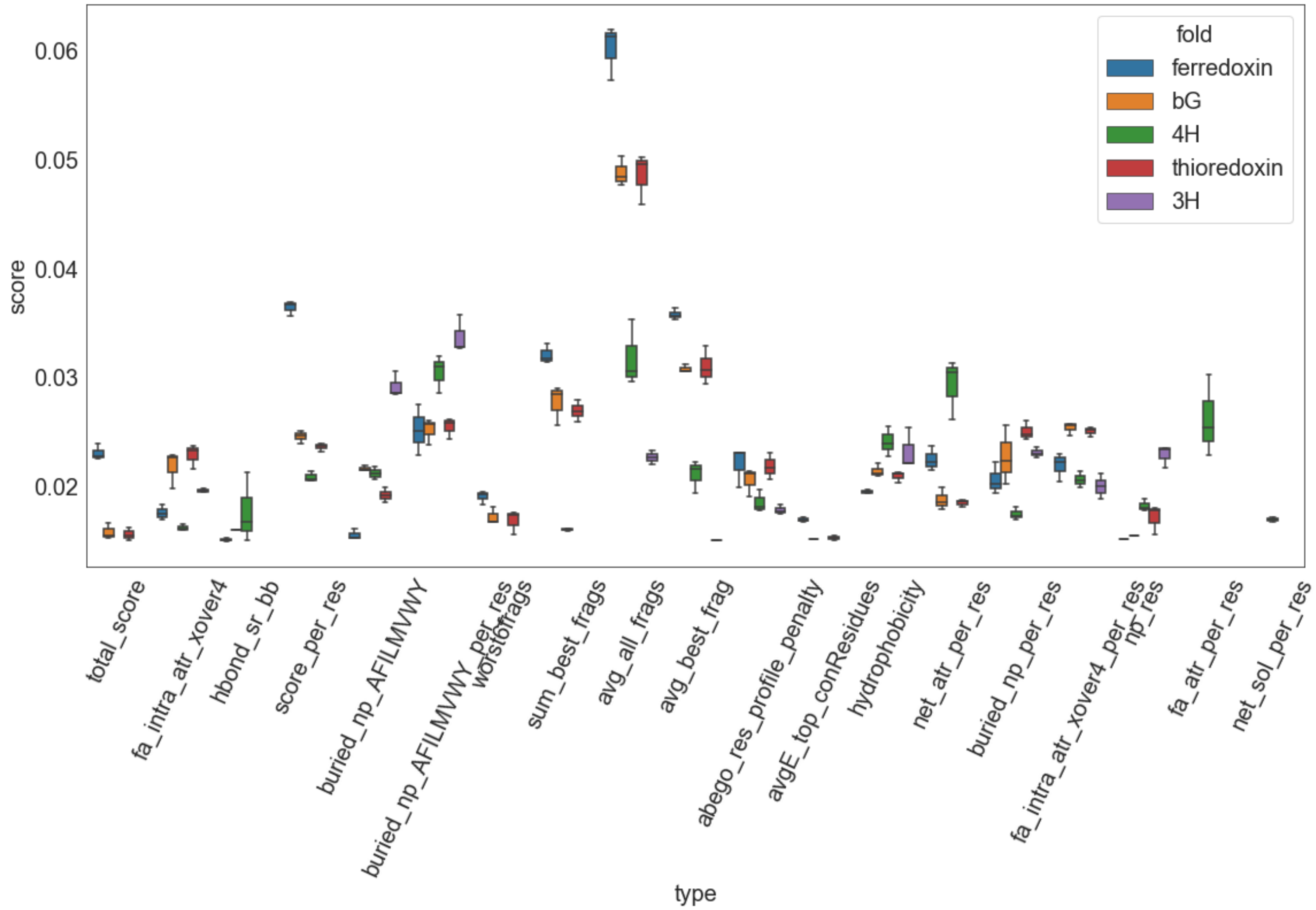

Supplementary Figure 22. Determining features and their contributions to stability predictions dependent on fold. Beta-sheet-containing proteins need an excellent fragment score, where the overall Rosetta score is the most predictive for 3H bundles. This graph represents the minimum, maximum, median (bar within the box), first quartile and third quartile in the data set.

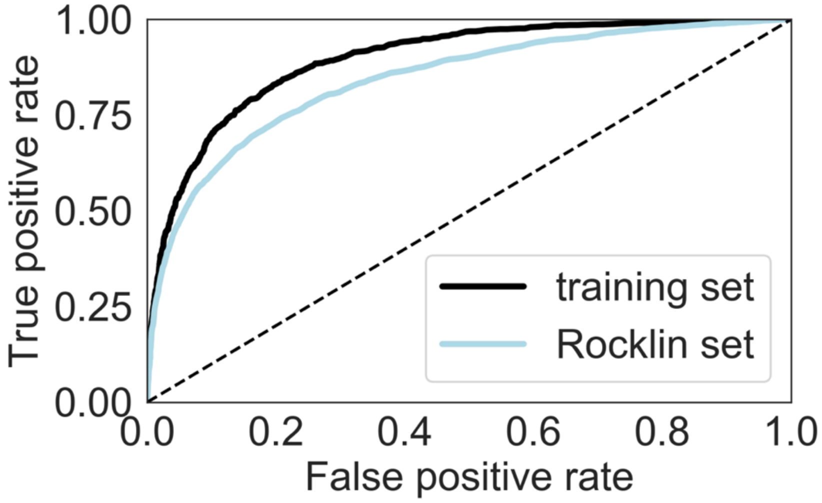

Supplementary Figure 23. Receiver operating characteristic curves (ROC) for stability prediction of an independent data set. Stability prediction for a previously *de novo* designed protein set, published by Rocklin et al.[3] showed an AUC of 0.84 (light blue) after training with the here reported protein scaffolds. To compare to our training set, 15% of the data points were taken out to evaluate the prediction.

Supplementary Tables

Supplementary Table S1. Characterization of individual proteins using size exclusion chromatography and circular dichroism (CD). For CD, the monomeric fractions for all proteins were measured, unless otherwise indicated. The oligomerization and its dominant species are indicated in bold (A=aggregate M=monomer, D=dimer).

name	chymo. stability	trypsin stability	stability score	expression yields	oligomeric state	folded	agreement with prediction
3hM_790	4.9	1.7	1.7	+++++	**M**	+	+
4hM_4108.2	4.2	2.1	2.1	+	**M**	+	+
4hM_424.1	1.9	3.7	1.9	++	A, D, **M**	+	+
4hM_3692	1.1	2	1.1	++++	**M**	+	+
coil_109.2	1.7	1.5	1.5	++++	**M**	+	+
bGM_166	1.5	1.7	1.5	+/-	A, **D**, M	+	-
bGM_518.1	2.1	1.8	1.8	++++	A, D, **M**	+	+
bGM_442	1.1	1.4	1.1	+	M	+	+
bGM_649	0.3	0.7	0.3	-	n/a	n/a	n/a
bGM_649.2	0.7	1.5	0.7	+/-	**A**, D, M	-	-
ferrC_1045	1.4	1.2	1.2	++	A, D, **M**	+	+
ferrM_4961	1.6	1.4	1.4	-	n/a	n/a	+
ferrM_5659.1	1.5	3.1	1.5	++++	**M**	+	+
thioFL24	1.2	-0.3	-0.3	+++	**M**	-	+
thioM_802	0.8	0.5	0.5	+++++	M	+	+
thioFL2_153	1.7	1	1	+	**A**, D, M	+	+
thioM_330	1.8	1.5	1.5	++++	**M**	+	+
f2_256	1.1	1.1	1.1	++	**A**, D, M	+	+
f2_118	1.1	1.1	1.1	++	A, **D**, M	+	+
f4m_280	1.7	1.5	1.5	++	A, **D**, M	+	+
f4m_256	1.9	1.3	1.3	++	A, **D**, M	-	-
f4m_256-dimer						+	n/a

Supplementary Table S2. Statistics summary for the 20 structure Thio-802 NMR ensemble

**NOE-based distance restraints**	
Intra-residue ($i = j$)	256
Sequential ($\|i-j\| = 1$)	311
Medium Range ($2 \leq \|i-j\| \geq 4$)	241
Long Range ($\|i-j\| \geq 5$)	329
Total	1137
**Hydrogen bond distance restraints**	0
**Dihedral angle restraints (□ and □)**	116
**Restraint Violations**	
NOE > 0.2 Å	0
Dihedral angle > 5º	0
**RMSD from the mean structure (Å)**	
Main chain (residues 2-63)	0.19 ± 0.08
Heavy atoms (residues 2-63)	0.77 ± 0.12
**RMSD from experimental restraints**	
NOE-based distance restraints (Å)	0.0279 ± 0.0003
Dihedral angle restraints (º)	1.04 ± 0.02
**RMSD from idealized covalent geometry**	
Bonds	0.0059 ± 0.00007
Angles	0.638 ± 0.006
Improper angles	0.531 ± 0.007
**Ramachandran analysis (PROCHECK-NMR), residues 2-63 (%)**	
Residues in most favored regions	94.0
Residues in additional allowed regions	5.9
Residues in generously allowed regions	0.1
**Protein Structure Validation Suite Analyses (version 1.5), ordered residues, 2-65 (Z-scores in parentheses)**	
PROCHECK G-factor (all dihedral)	-0.19 ± NA (-1.12)
PROCHECK G-factor (phi / psi)	0.01 ± NA (0.35)
Verify3D	0.19 ± 0.02 (-4.33)
ProsaII (-ve)	0.99 ± 0.04 (1.41)
*MolProbity clashscore	36.98 ± 2.89 (-4.82)
**MolProbity Clashscore Analysis (MolProbity server version 4.5.1), all residues**	
Clashscore	22.7 ± 2.1
*The MolProbity clashscores calculated by the PSVS server and the MolProbity server differ significantly. The clashscore returned by the PDB validation server is similar to the value from the MolProbity server (22 ± 2).	

Supplementary Methods

Examples, data files, and compute times

Files: XML for backbone generation of the different reported folds as well as design protocols, stability and Rosetta-related scores, reported new features and script to generate them are uploaded onto https://github.com/strauchlab/scaffold_design. Example folders with command line options (and scripts to generate them) together with expected output were also included on the github.

Compute times: On a single thread of an Intel® Xeon® Gold 6130 Processor (22M Cache, 2.10 GHz) it takes about 10 min – 2.5 h to compute a backbone structure (depending on the fold), if the parameters are suitable for the desired fold. For the sequence design of the computed backbone structures, it takes about 18 min – 45 min per decoy on a single core. Training of the Random Forest Classifier and predictions take about 1 min on a comparable processor.

Rosetta Versions: Backbone generation was tested with the following version 2020.50.post.dev+978.master.edd2dcd21e3 edd2dcd21e3bfbf1eb00085360bb17d6015bbbe5 git@github.com:RosettaCommons/main.git 2021-02-16T11:40:43. Sequence design has been tested with version: 2018.39.post.dev+173.HEAD.ce9cb33 ce9cb339991a7e8ca1bc44efb2b2d8b0a3d557f8. This version was also used for rescoring the original decoys. Designed models in form of pdbs can be send upon request.

Fragment analysis

To evaluate agreement between sequence and structure for a given designed protein, we used Rosetta's standard fragment generation protocol[4] to select 200 fragments from natural protein crystal structures for each 9-residue-long segment of the designed protein. The fragments were chosen so that their sequence and secondary structure were as similar as possible to the sequence and *predicted* secondary structure of the designed protein segment (predicted using PSIPRED). Geometric similarity was quantified as the average RMSD of all 200 fragments at all positions (the "avg_all_frags" metric described further below*: Definition of scoring metrics*). Other measures of agreement are also described in that section.

Adjustment of trypsin and chymotrypsin based on the "stability ladder"

Using the "stability ladder" of previously measured stability scores for our 5 proteins, we adjusted the chymotrypsin values by a factor 3.5 to reproduce the previously reported stability data. A linear relationship was assumed. The stability cutoff was determined by plotting and fitting stability scores of the 2,300 random sequences.

Definition of scoring metrics

<u>Simple sequence and topological properties:</u>

description: the design name
sequence: the design sequence
dssp: the design secondary structure, according to the DSSP algorithm
n_res: the number of residues in the design
nres_helix: the number of helical residues in the design, according to DSSP
nres_sheet: the number of beta strand residues in the design, according to DSSP
nres_loop: the number of loop residues in the design, according to DSSP
frac_helix: nres_helix / n_res
frac_sheet: nres_sheet / n_res
frac_loop: nres_loop / n_res
n_charged: the count of D, E, K, and R residues in the designed sequence, plus one-half the number of H residues.

netcharge: the net charge on the design, assuming a charge of +1 on R and K, +0.5 on H, and -1 on D and E.
AlaCount: the count of Ala residues in the design
n_hydrophobic: the count of A, F, I, L, M, V, W, and Y residues in the design
n_hydrophobic_noA: the count of F, I, L, M, V, W, and Y residues in the design

Rosetta energy terms:
fa_atr, fa_dun_dev, fa_dun_rot, fa_dun_semi, fa_elec, fa_intra_atr_xover4, fa_intra_elec, fa_intra_rep, fa_intra_sol_xover4, fa_intra_rep_xover4, fa_intra_sol_xover4, fa_rep, fa_sol, hbond_bb_sc, hbond_lr_bb, hbond_sc, hbond_sr_bb, lk_ball, lk_ball_bridge, lk_ball_bridge_uncpl, lk_ball_iso, omega, p_aa_pp, pro_close, rama_prepro, ref, ss_sc, total_score, yhh_planarity: all the scores in the Rosetta full-atom energy function

Simple combinations of Rosetta energy terms:
score_per_res: total_score / n_res
fa_atr_per_res: fa_atr / n_res
fa_rep_per_res: fa_rep / n_res
hbond_lr_bb_per_res: hbond_lr_bb / n_res
hbond_lr_bb_per_sheet: hbond_lr_bb / nres_sheet
hbond_sr_bb_per_helix: hbond_sr_bb / nres_helix
net_atr_per_res: (fa_atr + fa_rep) / n_res
net_sol_per_res: (fa_sol + fa_elec) / n_res
net_atr_net_sol_per_res: net_atr_per_res + net_sol_per_res

Rosetta filters:
See https://www.rosettacommons.org/docs/latest/scripting_documentation/RosettaScripts/Filters/Filters-RosettaScripts for all documentation.
cavity_volume: void volume inside the designed structure, in Å^3, computed with CavityVolume filter
degree: average number of residues in a 9.5 Å sphere around each residue, computed with AverageDegree filter
contact_all: number of sidechain carbon-carbon contacts in the designed structure, computed with AtomicContactCount filter
exposed_hydrophobics: exposed nonpolar surface area of the designed structure, in Å^2, computed using TotalSasa filter, set to compute hydrophobic-only SASA
exposed_polars: exposed polar surface area of the designed structure, in Å^2, computed using TotalSasa filter, set to compute polar-only SASA
exposed_total: total exposed surface area of the designed structure, in Å^2, computed using TotalSasa filter
fxn_exposed_is_np: exposed_hydrophobics / exposed_total
holes: a normalized measure of the void volume inside the designed structure, computed with Holes filter
helix_sc: the average shape complementarity of each helical secondary structure element with the rest of the structure, computed using SSShapeComplementarity filter, set to evaluate helices only
loop_sc: the average shape complementarity of each loop element with the rest of the structure, computed using SSShapeComplementarity filter, set to evaluate loops only
mismatch_probability: the geometric average probability (across all positions in the design) that the designed residues will *not* adopt their designed secondary structures, as calculated by the PSIPRED algorithm from the designed sequence. Computed using the SSPrediction filter.

pack: a normalized measure of packing density, computed using PackStat filter
unsat_hbond: number of buried, unsatisfied hydrogen bonding atoms, computing using
ss_sc: the average shape complementarity of each helical or loop element with the rest of the structure, computed using SSShapeComplementarity filter
BuriedUnsatHbonds filter
unsat_hbond2: number of buried, unsatisfied hydrogen bonding atoms, computing BuriedUnsatHbonds2 filter

<u>Custom metrics computed in Rosetta:</u>
These metrics are not built-in Rosetta filters, but are computed within the Rosetta software
buried_np: buried nonpolar surface area in the designed structure on all amino acids, computed using version1 definitions of total nonpolar surface area per residue
buried_np_AFILMVWY: buried nonpolar surface area in the designed structure on nonpolar amino acids (AFILMVWY), computed using version2 definitions of total nonpolar surface area per residue
buried_np_AFILMVWY_per_res: buried_np_AFILMVWY / n_res
buried_np_per_res: buried_np / n_res
buried_minus_exposed: buried_np - exposed_hydrophobics
buried_over_exposed: buried_np / exposed_hydrophobics
exposed_np_AFILMVWY: exposed nonpolar surface area in the designed structure on nonpolar amino acids (AFILMVWY)
one_core_each: the fraction of secondary structure elements (helices and strands) with one large hydrophobic residue (FILMVYW) at a position in the core layer of the designed structure
two_core_each: the fraction of secondary structure elements (helices and strands) with two large hydrophobic residues (FILMVYW) at positions in the core layer of the designed structure
ss_contributes_core: the fraction of secondary structure elements (helices and strands) with one large hydrophobic residue (FILMVYW) at a position in either the core or interface layer of the designed structure
res_count_core_SASA: the number of residues in the core layer of the designed structure, with layers defined using solvent accessible surface area-based criteria
res_count_core_SCN: the number of residues in the core layer of the designed structure, with layers defined using sidechain neighbors-based criteria
percent_core_SASA: res_count_core_SASA / n_res
percent_core_SCN: res_count_core_SCN / n_res

<u>Custom metrics computed using external scripts as described in Rocklin et al. :</u>
abego_res_profile: Each position *i* in the designed structure can be classified by its ABEGO type, and the ABEGO types of positions *i*-1, *i*, and *i*+1 form a triad that defines the three-residue local structure at a coarse level. The abego_res_profile metric is the sum over all positions *i* in the designed structure of log ((p_aa | abego triad) / (p_aa)), where (p_aa | abego triad) is the frequency of the designed amino acid (from position *i*) in regions of natural proteins sharing the same ABEGO triad as the designed region centered on position *i*, and p_aa is the overall frequency of the designed amino acid at position *i*. At each position, this score is positive when the designed amino acid is overrepresented (compared with its normal frequency) in regions of natural proteins with the same local ABEGO triad structure as the designed region, and the score is negative when the designed amino acid is underrepresented in regions of natural proteins with the same local ABEGO triad structure.
abego_res_profile_penalty: Same as abego_res_profile, except summing over only positions with negative abego_res_profile scores (positions where the designed residue is typically underrepresented in the local structure).

contig_not_hp_avg: average size of the contiguous (in primary sequence) regions of the designed sequence lacking a large hydrophobic residue (FILMVWY)
contig_not_hp_norm: contig_not_hp_avg / (n_res / (1 + n_hydrophobic_noA))
contig_not_hp_max: the size of the largest contiguous region (in primary sequence) in the designed sequence containing no large hydrophobic residues (FILMVWY)
contig_not_hp_internal_max: the size of the largest contiguous region (in primary sequence) in the designed sequence containing no large hydrophobic residues (FILMVWY), excluding the regions between the first and last large hydrophobic residues and the termini
hphob_sc_contacts: the total number of sidechain-sidechain contacts between large hydrophobic residues (FILMVWY) in the designed structure
hphob_sc_degree: hphob_sc_contacts / n_hydrophobic_noA
largest_hphob_cluster: the size of the largest group of large hydrophobic residues (FILMVWY) that are all connected by at least one contact to each other in the designed structure
n_hphob_clusters: the number of disconnected groups of large hydrophobic residues (FILMVWY), where a group is defined as residues that contact each other in the designed structure but do not contact residues outside of the group
hydrophobicity: total hydrophobicity of the designed sequence, using the amino acid hydrophobicity scale from.

The column headers are annotated below in *Definition of scoring metrics*.

Fragment quality analysis:
Fragments were chosen for each designed protein using the standard Rosetta fragment generation protocol, which uses the designed sequence and PSIPRED-predicted secondary structure[5] as input. These metrics quantify the geometric agreement between the selected 9-mer fragments and the corresponding 9-mer segments of the designs (200 9-mer fragments are chosen per designed segment).
avg_all_frags: the average RMSD of all selected fragments to their corresponding segments of the designs, in Å. (200 × (n - 8) fragments in total)
avg_best_frags: the average RMSD of the lowest-RMSD fragment for each designed segment, in Å. (n - 8 fragments in total)
sum_best_frags: the sum of the RMSDs of the lowest-RMSD fragment for each designed segment. (n - 8 fragments in total)
worstfrag: among the set of fragments that are the lowest-RMSD fragments for their positions, the highest RMSD found
worst6frags: among the set of fragments that are the lowest-RMSD fragments for their positions, the sum of the RMSDs of the six highest RMSD fragments

Feature expansion and RandomForest model
Designs were analyzed using previous metrics and new features that combine connectivity and energetic terms which can be found in the score files as well as the jupyter notebook for predictions. Additionally, to previously reported score terms[3] we integrated the following new terms:
most_conRE: takes the Rosetta residue energy of the most connected residues as measured by how many amino acids are within 6 Å of the most connected residue.
most_conREn: takes the Rosetta residue energy of the most connected residues as measured by how many amino acids are within 6 Å of the most connected residue and then normalizes based on the number of neighbors
graph_density: measured the graph density of the contacting residues within the given protein.
bad_hub_penalty: extra penalty if well connected residue does not score well

highly_conREsum: total energy sum of the 4 most connected residues.
highly_conREsum_pres: highly_conREsum normalized by number of total residues in protein
highly_conREsumNorm: sum of residues energies of the top connected residues after normalization to total score of the protein
highly_conREsumNorm_pres: highly_conREsumNorm divided by total residues.
avgE_top_conResidues: average energy to top connected residues
avgE_top_conResiduesNorm: avgE_top_conResidues / total residues
avg_con: average connectivity within 6 Å
median_con: median connectivity
max_con: highest number of neighbors within 6 Å
num_bad_res: number of low scoring residues within the top connected residues.

Thio-802 NMR structure analysis

The amino acid sequence of the Thio-802 design used for structure determination by NMR spectroscopy included an N-terminal methionine residue and a C-terminal Leu-Glu linker followed by a six-residue histidine affinity tag. Including an N-terminal Met residue that could not be observed by NMR, the resulting sequence is 71 amino acids (N-terminal Met, 62 residue designed protein, C-terminal Leu-Glu and six His affinity tag). So, with regard to residue numbering, residues 1-62 of the designed protein correspond to residues 2-63 of the construct used for NMR.

Following refinement, the 20 Thio-802 structures, determined using NMR spectroscopy, with the lowest overall energies were chosen for final analysis. A summary of restraint information for the structure calculations and measures of overall structural quality for this 20-member ensemble is presented in Table S2. The overall RMSD for the main chain atoms of the ensemble is low, indicating good agreement for the atomic coordinates of the main chain for the members of the ensemble. The heavy atom RMSD is also low. There are no significant experimental restraint violations and 94% of the main chain dihedral angles are in the most favored regions of the Ramachandran space. These are all reliable indicators of high-quality structures. This ensemble comprised the PDB deposition (7LDF).

A stereo view of the superposition of the main chain ribbon of the 20 members of the structural ensemble is shown in Figure S18. For most regions of the structure, the near perfect superposition of the main chains reflects the low RMSD and low distance displacements (Table S2, Fig. S18). The exceptions are the two regions noted above. A topology diagram based on the output of the HERA program[7] and various views of the ribbon diagram of the lowest energy structure of the NMR ensemble is also shown.

NMR analysis of additional designed proteins

1D, ^{1}H NMR spectra of four of the designed proteins were collected to assess their folding and structure. The full spectra are shown in Figure S11, as are expansions of the amide/aromatic (downfield) regions. Three of the four are all helical (the proteins named coil 109.2, 4hM_3692, and 4h_4108.2), and the fourth (bGM_442) is comprised of both helix and beta sheet elements. In all cases, the signals in the NMR spectra are sharp, and the chemical shift dispersion is large, indicative of the tertiary structure of folded proteins. In the spectrum of bGM_442, the group of signals at ~5.0-5.4 ppm, just downfield of the water signal (4.76 ppm), is diagnostic of alpha hydrogens in regions of beta sheet structure, and this confirms the presence of beta sheet elements in this protein. Such signals are not present in the spectra of the all-helical proteins, as expected. The signals downfield of 10 ppm for the all-helical proteins are

due to the indole amine hydrogens in tryptophan side chains (there are no tryptophan residues in the bGM_442 protein). In the amide region for bGM_442, the chemical shifts of the signals are noticeably more dispersed than for the helical proteins, which is characteristic of proteins that include beta sheet secondary structure versus those with only helical secondary structure. Finally, for all of the proteins, signals upfield of ~0.9 ppm indicate methyl groups shifted upfield from random coil values due to magnetic anisotropy effects resulting from proximity to rings or carbonyl groups as a result of folding (tertiary structure)[8].

References

1. Drew, E.D. & Janes, R.W. PDBMD2CD: providing predicted protein circular dichroism spectra from multiple molecular dynamics-generated protein structures. *Nucleic Acids Res* **48**, W17-W24 (2020).
2. Rocklin, G.J. et al. Global analysis of protein folding using massively parallel design, synthesis, and testing. *Science* **357**, 168-175 (2017).
3. Rohl, C.A., Strauss, C.E., Misura, K.M. & Baker, D. Protein structure prediction using Rosetta. *Methods in enzymology* **383**, 66-93 (2004).
4. Jones, D.T. Protein secondary structure prediction based on position-specific scoring matrices. *Journal of molecular biology* **292**, 195-202 (1999).
5. Berjanskii, M.V. & Wishart, D.S. A simple method to predict protein flexibility using secondary chemical shifts. *J Am Chem Soc* **127**, 14970-14971 (2005).
6. Hutchinson, E.G. & Thornton, J.M. HERA--a program to draw schematic diagrams of protein secondary structures. *Proteins* **8**, 203-212 (1990).
7. Wuthrich, K. *NMR of Proteins and Nucleic Acids* (Wiley, New York), Chapter 3, pp. 26-39 (1986).
