# Supplementary material for: Sampling of structure and sequence space of small protein folds
Source: Nat Commun. 2022 Nov 22;13:7151. doi: 10.1038/s41467-022-34937-8 (PMC9684540; doi:10.1038/s41467-022-34937-8)
Supplement: Supplementary file 2 — Reporting Summary [file 41467_2022_34937_MOESM2_ESM.pdf]

nature portfolio

Corresponding author(s): Strauch, EM

Last updated by author(s): Nov 2, 2022

Reporting Summary

Nature Portfolio wishes to improve the reproducibility of the work that we publish. This form provides structure for consistency and transparency in reporting. For further information on Nature Portfolio policies, see our Editorial Policies and the Editorial Policy Checklist.

Statistics

For all statistical analyses, confirm that the following items are present in the figure legend, table legend, main text, or Methods section.

n/a	Confirmed	
☐	☒	The exact sample size (n) for each experimental group/condition, given as a discrete number and unit of measurement
☐	☒	A statement on whether measurements were taken from distinct samples or whether the same sample was measured repeatedly
☒	☐	The statistical test(s) used AND whether they are one- or two-sided<br>*Only common tests should be described solely by name; describe more complex techniques in the Methods section.*
☒	☐	A description of all covariates tested
☒	☐	A description of any assumptions or corrections, such as tests of normality and adjustment for multiple comparisons
☐	☒	A full description of the statistical parameters including central tendency (e.g. means) or other basic estimates (e.g. regression coefficient) AND variation (e.g. standard deviation) or associated estimates of uncertainty (e.g. confidence intervals)
☒	☐	For null hypothesis testing, the test statistic (e.g. F, t, r) with confidence intervals, effect sizes, degrees of freedom and P value noted<br>*Give P values as exact values whenever suitable.*
☒	☐	For Bayesian analysis, information on the choice of priors and Markov chain Monte Carlo settings
☒	☐	For hierarchical and complex designs, identification of the appropriate level for tests and full reporting of outcomes
☒	☐	Estimates of effect sizes (e.g. Cohen's d, Pearson's r), indicating how they were calculated

Our web collection on statistics for biologists contains articles on many of the points above.

Software and code

Policy information about availability of computer code

Data collection	Fluorescence activated cell sorting was performed using a Sony SH800 and their software (2017 version), NGS data was obtained through Illumina NextSeq (500/550 Midi kit). SEC data was obtained using an Akta PURE together with a S75 increase column 30/100. CD data was obtained through Aviv or Olis. Each data point was recorded as at least triplicates.
Data analysis	The software to generate the new scaffolds is part of the Rosetta macromolecular software code. We utilized version 2018.39.post.dev +173.HEAD.ce9cb33 ce9cb339991a7e8ca1bc44efb2b2d8b0a3d557f8 for our design but also tested version 2020.50.post.dev +978.master.edd2dcd21e3 edd2dcd21e3bfbf1eb00085360bb17d6015bbbe5 git@github.com:RosettaCommons/main.git 2021-02-16T11:40:43 to ensure the backbone generation code would work. To efficiently use it, we developed an interface with RosettaScripts and provide XML scripts for backbone design and sequence design. These are posted under our https://github.com/strauchlab/scaffold_design account. For flow cytometry, we used FlowJo version 8 and 10.8. For Next-generation sequencing, we used an Illumina NextSeq and previously published custom python code for its analysis (Rocklin et al. DOI: 10.1126/science.aan0693) and data fitting. Comparison of stability scores was done through jupyter notebooks which are also part of the github account: https://github.com/strauchlab/scaffold_design. SEC data was plotted using Excel (version 16.54). Circular dichroism raw data through the Aviv instrument is text based and can be plotted with a simple python or simply using Excel. Olis CD data needs to be decoded. A jupyter notebook (Anaconda2 package, Python 2.7.16 |Anaconda, Inc.| (default, Sep 24 2019) that can plot CD data and decode Olis data has been posted into the associated github account (https://github.com/strauchlab/scaffold_design).

For manuscripts utilizing custom algorithms or software that are central to the research but not yet described in published literature, software must be made available to editors and reviewers. We strongly encourage code deposition in a community repository (e.g. GitHub). See the Nature Portfolio guidelines for submitting code & software for further information.

Data

Policy information about availability of data

All manuscripts must include a data availability statement. This statement should provide the following information, where applicable:

- Accession codes, unique identifiers, or web links for publicly available datasets
- A description of any restrictions on data availability
- For clinical datasets or third party data, please ensure that the statement adheres to our policy

The NMR structural ensemble has been deposited to the PDB and will be released upon publication. Computed scores and stability scores, sequencing count summary can be found in the listed github repository. Models of designed proteins and next-generation sequencing can be sent upon request.

Field-specific reporting

Please select the one below that is the best fit for your research. If you are not sure, read the appropriate sections before making your selection.

☒ Life sciences ☐ Behavioural & social sciences ☐ Ecological, evolutionary & environmental sciences

For a reference copy of the document with all sections, see nature.com/documents/nr-reporting-summary-flat.pdf

Life sciences study design

All studies must disclose on these points even when the disclosure is negative.

Sample size	For each FACS sort, we sorted 10 mio cells. Given a diversity of 31,500 seuqences, we oversampled the library size by 1000 fold which should that each design is seen as previously reported (Dou et al DOI: DOI https://doi.org/10.1039/C9ME00118B)<br>A forward and reverse read (PE150) was utilized to ensure sequences were as intended.
Data exclusions	Data exclusion for EC50 values was based on confidence intervals obtained during fitting process (Rocklin et al. DOI: 10.1126/science.aan0693). Data outside the margin was not considered.
Replication	We included additional selections for the protease digestion (as recorded under the experiments.csv) beyond the originally reported assay. Proteins were expressed, purified and characterized at least twice at different days. SEC and CD was at least done in duplicates. CD measurements was based on at least 3 replications. AUCs obtained through predictions were based on triplicates. All replicates were successful.
Randomization	The sequence of 2,300 randomly picked designed scaffolds were randomized in their sequence order and used as controls. Sequences are provide, they start with rand_ followed by the name of the original scaffold protein name.
Blinding	For cell sorting, control was not blinded, but all other samples were sorted in random order (blinded samples).

Reporting for specific materials, systems and methods

We require information from authors about some types of materials, experimental systems and methods used in many studies. Here, indicate whether each material, system or method listed is relevant to your study. If you are not sure if a list item applies to your research, read the appropriate section before selecting a response.

Materials & experimental systems

n/a	Involved in the study
☐	☒ Antibodies
☐	☒ Eukaryotic cell lines
☒	☐ Palaeontology and archaeology
☒	☐ Animals and other organisms
☒	☐ Human research participants
☒	☐ Clinical data
☒	☐ Dual use research of concern

Methods

n/a	Involved in the study
☒	☐ ChIP-seq
☐	☒ Flow cytometry
☒	☐ MRI-based neuroimaging

Antibodies

Antibodies used	anti-C-Myc (Chicken) conjugated to FITC was purchased from ICL Lab, catalog number: CMYC-45F
Validation	The antibody was used for monitoring display of designed proteins on the surface of yeast cells. It is commercially available: https://www.icllab.com/anti-c-myc-antibody-chicken-fitc-conjugated-cmyc-45f.html. Cells were treated with proteases which should release the myc-tag which is what we monitored. The following most recent publications have used this antibody from ICL:

Greaney AJ. et al.
Complete Mapping of Mutations to the SARS-CoV-2 Spike Receptor-Binding Domain that Escape Antibody Recognition,
Cell Host & Microbe,Volume 29, Issue 1,2021,Pages 44-57.e9,ISSN 1931-3128,
https://doi.org/10.1016/j.chom.2020.11.007.

Starr TN, Greaney AJ, Hilton SK. et al.
Deep Mutational Scanning of SARS-CoV-2 Receptor Binding Domain Reveals Constraints on Folding and ACE2 Binding.
Cell. 2020;182(5):1295-1310.e20. doi:10.1016/j.cell.2020.08.012

Eukaryotic cell lines

Policy information about cell lines

Cell line source(s)	EBY100 Saccharomyces cerevisiae, University of Washington
Authentication	Cell line was not authenticated
Mycoplasma contamination	N/A
Commonly misidentified lines (See ICLAC register)	N/A

Flow Cytometry

Plots

Confirm that:

- [x] The axis labels state the marker and fluorochrome used (e.g. CD4-FITC).
- [x] The axis scales are clearly visible. Include numbers along axes only for bottom left plot of group (a 'group' is an analysis of identical markers).
- [x] All plots are contour plots with outliers or pseudocolor plots.
- [x] A numerical value for number of cells or percentage (with statistics) is provided.

Methodology

Sample preparation	EBY100 yeast cell cultures were induced for 16-18 h at 30ºC in SGCAA23. Induced cells washed once with 20 mM NaPi 150 mM NaCl pH 7.4 (PBS), normalized to 1 mL at O.D. 1 (12-15 mio.M cells), washed and resuspended in 250 µL buffer PBS for trypsin reactions, or 20 mM Tris 100 mM NaCl pH 8.0 with (TBS) for chymotrypsin reactions). Protolysis was initiated by adding 250 µL of room temperature protease in buffer (PBSF or TBSF) followed by vortexing and incubating the reaction at room temperature (proteolysis reactions took place at cell O.D. 2).<br>The library was assayed at five protease concentrations over different rounds of sequential selection rounds as summarized in the experiments.csv file. For trypsin digestions we used 0.07 µM, 0.21 µM, 0.64 µM, 1.93 µM, and 5.78 µM protease; chymotrypsin assays used 0.08 µM, 0.25 µM, 0.74 µM, 2.22 µM, and 6.67 µM protease.<br>Cells were labeled with anti-C-Myc conjugated to FITC antibody (chicken) - 2 uL in 100 uL and then washed with ice cold 1 mL PBSF before sorting.
Instrument	SONY SH800SAC (2017 version)
Software	Sony proprietary and FlowJo v 8.2 and 10.8 for Mac
Cell population abundance	FSC and SSC was used focused on yeast cells; populations were above 85%
Gating strategy	Yeast cells were gated based on SSC and FSC; more than 85% was found within specified population. Cells not subjected to proteases were labeled with anti-C-Myc-FITC antibody. Two distinct populations were visible for FL-1 (FITC); one fluorescent and one not. For all selections, a gate taking the fluorescent population of that control as a standard was used. The FL-1 was > 2000 for the selected cells and gating is illustrated in Fig. S6.

- [x] Tick this box to confirm that a figure exemplifying the gating strategy is provided in the Supplementary Information.
